# Getting the Manifold Right: The Crucial Role of Orbital Resolution in DFT+*U* for Mixed d–f Electron Compounds

**DOI:** 10.1021/acs.jctc.5c01406

**Published:** 2026-01-07

**Authors:** Kinga Warda, Eric Macke, Iurii Timrov, Lucio Colombi Ciacchi, Piotr M. Kowalski

**Affiliations:** † Forschungszentrum Jülich GmbH, 49557 Institute of Energy Technologies, Theory and Computation of Energy Materials (IET-3), 52425 Jülich, Germany; ‡ Jülich Aachen Research Alliance JARA Energy and Center for Simulation and Data Science (CSD), 52428 Jülich, Germany; § Chair of Theory and Computation of Energy Materials Faculty of Georesources and Materials Engineering RWTH Aachen University, 52062 Aachen, Germany; ∥ Faculty of Production Engineering, Bremen Center for Computational Materials Science and MAPEX Center for Materials and Processes, Hybrid Materials Interfaces Group, 9168 University of Bremen, D-28359 Bremen, Germany; ⊥ U Bremen Excellence Chair, 28334 University of Bremen, D-28359 Bremen, Germany; # PSI Center for Scientific Computing, Theory, and Data, Paul Scherrer Institute, 5232 Villigen PSI, Switzerland

## Abstract

Accurately modeling compounds with partially filled d and f shells remains a hard challenge for density-functional theory, due to large self-interaction errors stemming from local or semilocal exchange-correlation functionals. Hubbard *U* corrections can mitigate such errors, but are often detrimental to the description of hybridized states, leading to spurious force contributions and wrong lattice structures. Here, we show that careful disentanglement of localized and delocalized states leads to accurate predictions of electronic states and structural distortions in ternary monouranates (AUO_4_, where A represents Mn, Co, or Ni), for which standard *U* corrections generally fail. Crucial to achieving such accuracy is a minimization of the mismatch between the spatial extension of the projector functions and the true coordination geometry. This requires Wannier-like alternatives to atomic-orbital projector functions, or corrections of Hubbard manifolds exclusively comprised of the most localized A-3d, U-5f and O-2p orbitals. These findings open up the computational prediction of fundamental properties of actinide solids of critical technological importance.

## Graphical abstract

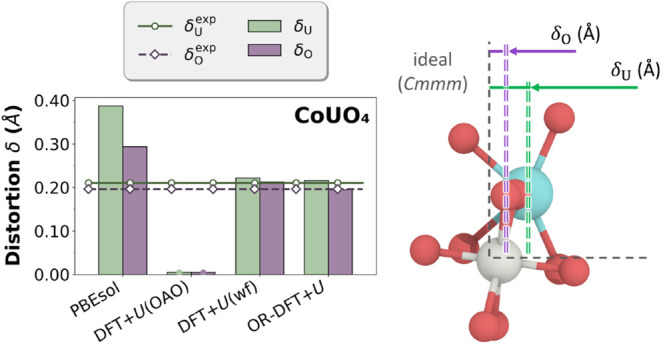

## 1 Introduction

Computational investigations play a crucial role in the research workflow on energy materials.^1^ This is especially the case for nuclear materials, where direct measurements are often limited to surrogate systems in which hazardous actinide elements are replaced by harmless lanthanides.^2,3^ However, the key elements that determine the performance of electrocatalysts, batteries or nuclear fuel and waste, featuring partially filled d and f shells—including transition metals and actinides—pose a challenge for density-functional theory (DFT), the main tool for prediction of the structure and electronic properties of functional materials. This is because the (typically localized) d and f electrons are particularly prone to spurious self-interaction errors (SIEs) that arise from the inexact cancellation of the Hartree term by approximate exchange-correlation (xc) functionals.^4–6^ As a result, several features of exact DFT are not correctly reproduced by LDA and GGA-type xc functionals: the piecewise linearity (PWL) of the total energy with respect to fractional addition or removal of charge,^4,7,8^ the existence of a derivative discontinuity,^9,10^ and the correct asymptotic decay of the Kohn–Sham (KS) potential.^11^ These shortcomings ultimately compromise the prediction of several fundamental properties, including lattice symmetries, bond strengths and band gaps. For instance, GGA functionals predict UO_2_—the main form of nuclear fuel—to be metallic, whereas it is in fact a Mott insulator with a band gap of ∼2 eV.^12^

One way to mitigate SIEs is the DFT+*U* framework.^13–16^ In this approach, a corrective on-site term scaled by a Hubbard parameter *U* is applied to a predefined electronic subspace dubbed Hubbard manifold in order to remove (locally) the spurious quadratic deviations from PWL,^17^ while also reintroducing a derivative discontinuity. Note that due to its history as a remedy to the unsatisfactory performance of (semi)­local functionals in predicting the electronic structure of strongly correlated materials, DFT+*U* is often understood as a method that corrects for strong electronic correlations not well captured by DFT; however, this view has been increasingly challenged.^17–19^ In the commonly used approximation, the Hubbard manifold encompasses the entirety of valence d and f shells, and all magnetic quantum orbitals of these shells are corrected using a shell-averaged scalar Hubbard *U* parameter. This approximation implicitly relies on the assumption that all d and f states are localized and therefore display only weak interactions with the surrounding electron bath. To apply the correction, the orbital occupations within the Hubbard manifold are determined by projecting the Kohn–Sham wave functions onto a suitable mathematical representation, henceforth referred to as Hubbard projectors. A straightforward and widely used choice for this representation consists in atomic-like orbitals parametrized based on solutions of radial Schrödinger equations for isolated atoms. Shell-averaged DFT+*U* with such atomic orbital projectors has been successfully applied to study the electronic and ionic structures of a variety of solids bearing lanthanides and actinides.^20–23^

Geological disposal of nuclear waste is a challenge faced by many countries that utilize nuclear technology.^24^ A particular concern is the formation of secondary phases between disposed UO_2_-based nuclear fuel, fission products, and near-field elements. A notable example of these secondary phases is provided by ternary monouranates AUO_4_, with A representing a bi- or trivalent metal.^25^ Such materials are often studied using DFT+*U* schemes. However, for AUO_4_ compounds with A = Ni, Mg, Co, Mn, the shell-averaged approach was shown to incorrectly stabilize a higher-symmetry *Cmmm* structure instead of the experimentally observed *Ibmm* phase.^26^ The failure of shell-averaged DFT+*U* was attributed to a significant overestimation of d and f orbital occupancies due to the use of atomic orbital Hubbard projectors, which led to large artificial Hubbard energy contributions.^26^ To demonstrate this, the authors set the Hubbard energy term *E*_*U*_ to zero, and with this recovered the experimentally observed *Ibmm* structures. Only for A = Cd did the *Cmmm* structure remain stable, again in line with experimental results. Improvements similar to the *E*_*U*_ = 0 procedure were obtained by replacing the atomic-like Hubbard projectors with Wannier-type ones (hereafter denoted as the DFT+*U*(WF) method). Wannier-type Hubbard projectors yield more realistic (i.e., closer to integer values) d and f occupations, so that the spurious Hubbard energy contributions vanish naturally. The DFT+*U*(WF) method has also proven valuable in other systems containing actinides and transition-metal (TM) atoms, offering substantial improvements in the predicted electronic structure, thermodynamic properties, and X-ray spectra.^22,27–30^

Despite these advantages, DFT+*U*(WF) comes with practical limitations: the construction of Wannier functions is sensitive to initialization choices (energy windows, number of bands, disentanglement procedure), and—depending on the type of Wannier function used—the direct evaluation of forces and stresses is not yet implemented in the codes. An alternative approach that also seeks to avoid the overcorrections of shell-averaged DFT+*U* is orbital-resolved DFT+*U* (OR-DFT+*U*).^19^ This method is grounded in the insight that the degree of electron localization (and thus the severity of SIEs) can vary significantly at the *intrashell* level, i.e., between different *nlm* orbitals of the same *nl* subshell.^31^ OR-DFT+*U* provides an *ad-hoc* solution that enables the definition of pinpoint Hubbard manifolds spanned by simple atomic orbital projectors for compounds where shell-averaged DFT+*U* fails (refs 19,32–35 report examples of such failures).

In this study, we apply various orbital-resolved Hubbard manifolds to the ternary TM monouranates β-NiUO_4_, CoUO_4_ and MnUO_4_, aiming to reproduce the experimentally observed structural distortions without imposing additional constraints on the Hubbard energy or introducing other empirical assumptions or modifications. All Hubbard *U* parameters are computed from first-principles using the linear-response constrained DFT approach (LR-cDFT).^17,33^ By testing different orbital-resolved Hubbard manifolds (determined through careful analyses of orbital occupations), we aim at identifying the root cause of the failure of shell-averaged DFT+*U* in these systems. Finally, we assess how the definition of the Hubbard manifold and the choice of projector functions influence the prediction of structural observables and clarify under which conditions OR-DFT+*U* can serve as a robust and practical alternative to the DFT+*U*(WF) framework for modeling d and f electron systems with strong covalent bonding.

## 2 Materials and Methods

### 2.1 Computational Details

DFT calculations were performed using the pw.x code contained in the Quantum ESPRESSO package.^36–38^ The calculation setup was chosen to match the computational setup used by Murphy et al.^26^ This included employing the PBEsol exchange-correlation functional^9^ and ultrasoft pseudopotentials (self-generated using the Vanderbilt code^39^) with a plane-wave energy cutoff of 50 Ry and a charge density cutoff of 200 Ry. Structural relaxations were carried out until the total energy and atomic forces converged below 10^–5^ Ry and 10^–4^ Ry/Bohr, respectively. All calculations were spin-polarized within the collinear approximation (assuming ferromagnetic order of the TM sites) and used a tight convergence threshold for electronic total-energy self-consistency of 10^–8^ Ry. Projected density of states (PDOS) calculations were performed with a Gaussian broadening of 0.0035 Ry. Moreover, the option diag_basis =.true. in the projwfc.x code was enabled to rotate atomic orbitals into the eigenbasis of the occupation matrix, thus aligning orbital projections with the local symmetry and facilitating a clearer identification of orbital character.^40^ An additional analysis of the electronic structure of NiUO_4_ employed the linear combination of fragment orbitals method^41^ as implemented in the LOBSTER tool,^42^ for which a single calculation had to be redone using PAW-type pseudopotentials (taken from the PSlibrary).

For all DFT+*U* calculations, we applied the formulation of Dudarev et al.^16^ that seeks to mitigate SIEs by enforcing (locally) PWL of the total energy with respect to fractional orbital occupations.^17,18^ Note that in this work, “DFT+*U*” refers exclusively to PBEsol+*U*. We used both shell-averaged and orbital-resolved Hubbard manifolds.^19,32,33^ The OR-DFT+*U* energy functional is given by

1 $$E_{\mathrm{DFT}+U}=E_{\mathrm{DFT}}+E_{U}$$

where

2 $$E_{U}=\sum_{I,\sigma }\sum_{i=1}^{2l+1}\frac{U_{i}^{I}}{2}\lambda_{i}^{I\sigma }\left(1-\lambda_{i}^{I\sigma }\right)$$

and the corresponding Hubbard potential is

3 $$V_{U}^{\sigma }=\sum_{I}\sum_{i=1}^{2l+1}U_{i}^{I}\left(\frac{1}{2}-\lambda_{i}^{I\sigma }\right)\left|\phi_{i}^{I}\right\rangle \left\langle \phi_{i}^{I}\right|$$

Here, *U*_*i*_^*I*^ is the Hubbard parameter corresponding to the (diagonal) orbital {ϕ_*i*_^*I*^} of atom *I* (the principal and angular quantum numbers *nl* are omitted for clarity), σ is the spin index, and λ_*i*_^*I*σ^ are the eigenvalues of the occupation matrix, computed by solving the eigenvalue problem ∑_*m*′_ *n*_*mm*′_^*I*σ^ ν_*m*′,*i*_^*I*σ^ = λ_*i*_^*I*σ^ ν_*m*,*i*_^*I*σ^, where ν^*I*σ^ are the corresponding eigenvectors and *n*_*m m*′_^*I*σ^ are the elements of the occupation matrix for the magnetic quantum numbers *m* and *m*′. In the shell-averaged case, both expressions simplify as *U*_*i*_^*I*^ = *U* ^*I*^ for *all i*. Orthogonalized atomic orbitals (OAO)­{φ_*m*_^*I*^} were employed as Hubbard projector functions. In eq 3, these orbitals are written in their diagonal basis {ϕ_*i*_^*I*^}, which can be back-transformed into the nondiagonal setting by substituting |ϕ_*i*_^Iσ^⟩= ∑*_m_* *ν*_*mi*_^*Iσ*^ |φ_*m*_^*I*^⟩. We highlight that the Hubbard projector functions are independent of spin; however, for each spin channel, they are rotated separately using the corresponding eigenvectors of the spin-resolved occupation matrix.

OAO are closely related to the nonorthogonalized projectors used by Murphy et al.,^26^ but prevent the double-counting of Hubbard corrections in regions where projector orbitals overlap.^43,44^ With these projector functions, the occupation matrix **n** is computed as

4 $$n_{mm^{\prime }}^{I\sigma }=\sum_{k,v}f_{k,v}^{\sigma }\left\langle \psi_{k,v}^{\sigma }\right|\varphi_{m\prime }^{I}\rangle \left\langle \varphi_{m}^{I}\right|\psi_{k,v}^{\sigma }\rangle$$

where *m* and *m*′ are magnetic quantum numbers, and ψ_**k**,v_^σ^ are the KS wave functions at point **k** with the band index *v* and spin σ (additional operators arise when using ultrasoft and PAW basis sets^44^). All on-site *U* parameters were evaluated from first-principles using the LR-cDFT approach introduced in ref 17 and extended to the orbital-resolved formalism in ref 19. It is important to note that shell-averaged Hubbard *U* values are not equivalent to the arithmetic mean of the orbital-resolved *U* parameters associated with a given shell.^19^ Instead, the shell-averaging is performed at level of the response to the perturbation, which frequently results in shell-averaged *U* values exceeding both the individual orbital-resolved values and their arithmetic mean.^33^ In the LR-cDFT calculations, perturbations of magnitude α = ± 0.05 eV were applied to the respective manifold of a single atom in a 2 × 2 × 2 supercell that was created to avoid unphysical interactions of the perturbed state with its periodic images. It is important to note that the Quantum ESPRESSO implementation of LR-cDFT ensures that only the DFT part of the linear response is assessed, as the potential derived from the Hubbard functional is kept fixed during perturbative calculations.^45,46^ The Brillouin zone was sampled using Γ-centered 4 × 4 × 4 and 2 × 2 × 2 Monkhorst–Pack grids for the unit cell and the supercell calculations, respectively. As the responses to perturbations depend on the electronic structure (which in turn depends on the ionic structure), calculated Hubbard parameters can change when transitioning from a DFT ground state to a DFT+*U* one. While there exist procedures that self-consistently derive the Hubbard parameters and optimize the ionic structure,^45,47^ we did not adopt such an approach here. Instead, all LR-cDFT calculations were carried out in a “one-shot” manner on top of DFT+*U* ground states obtained using an empirical guess for the *U* values: *U*_Ni–3d_ = *U*_Co–3d_ = 4.0 eV, *U*_Mn–3d_ = 2.0 eV, *U*_U–5f_ = 2.0 eV, and *U*_O–2p_ = 1.0 eV. This procedure was previously shown to yield Hubbard parameters typically within *δ U* = *U*_SC_ – *U* ≤ 0.1 eV (where *U*_SC_ is the self-consistent value), provided the input structure does not deviate strongly from the final relaxed one.^48^ We have confirmed that this rapid convergence also holds for the AUO_4_ compounds discussed in the following (Table S3).

### 2.2 Characterization of the Structural Distortions

The crystal structure of *Ibmm* ternary monouranates is similar to rutile (TiO_2_, space group *P*4_2_/*mnm*),^49^ featuring parallel one-dimensional chains of edge-sharing AO_6_ and UO_6_ octahedra that run along the [001] direction (Figure 1a). Each U atom is coordinated by two O1 atoms in a *trans* configuration and four equatorial O2 atoms, whereas the reverse arrangement occurs in the AO_6_ octahedra. A distinctive feature of the *Ibmm* structure are strongly hybridized uranyl O1=U=O1 moieties, which display significantly shorter bond lengths than the U–O2 bonds.^49^ These moieties are associated with structural distortions^50^—namely octahedral tilting (*θ*, Figure 1b) and off-centering distortions of uranium and oxygen atoms (*δ*, Figure 1c)—with respect to the higher-symmetry *Cmmm* structure of CdUO_4_. The tilting angle *θ* measures the deviation of the O2 atoms from collinearity with the *b*-axis and is given by

5 $$\theta =90^{{}^{\circ}}-\tan^{-1}\left[\frac{\left(2x-1\right)a}{2\mathrm{zc}}\right]$$

where *x* and *z* are the fractional coordinates of the O2 atoms, and *a* and *c* are lattice parameters. The uranium off-centering *δ*_U_ and the oxygen off-centering *δ*_O_ are defined as the displacements of the U and O2 atoms from the center of the adjacent AO_6_ octahedron, measured along the [100] direction (Figure 1c). Specifically, *δ*_U_ and *δ*_O_ correspond to the differences between the atomic coordinates of U and O2, and the coordinate of the octahedral center, respectively, all projected onto the [100] direction, capturing the structural distortion along the primary axis of asymmetry. Displacements along other directions are neglected.

**Figure 1:**
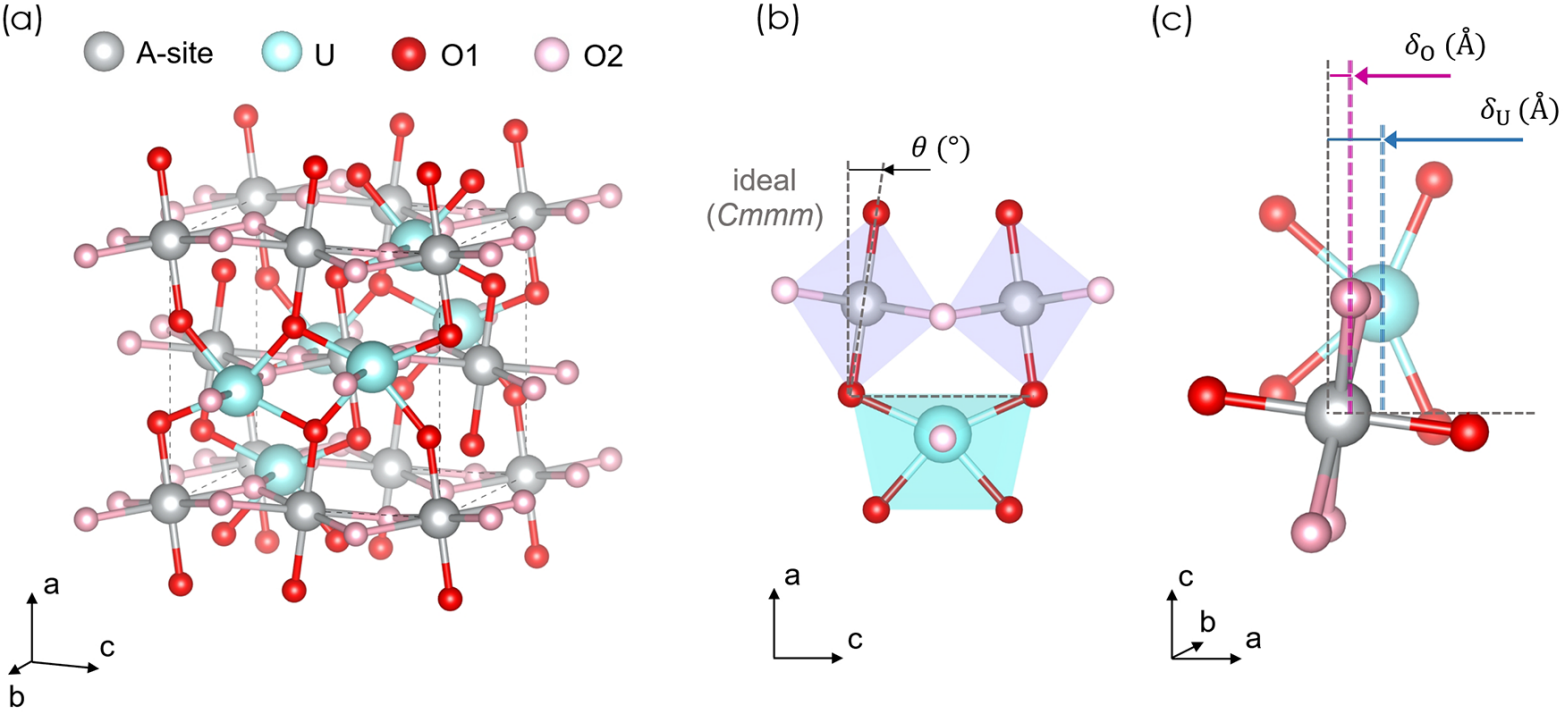
(a) Crystal structure of AUO_4_ in the orthorhombic *Ibmm* space group. Oxygen atoms O1 and O2 are shown in red and pink, respectively. (b) Schematic representation of the AO_6_ tilt angle *θ* around the *b*-axis, quantifying deviations from the idealized *Cmmm* symmetry. The UO_6_ octahedra is shown in cyan and the AO_6_ octahedra in purple. (c) The uranium off-centering *δ*_U_ and axial oxygen off-centering *δ*_O_, measured relative to the ideal *Cmmm* atomic positions. The high-symmetry *Cmmm* reference structure is indicated by dashed gray lines.

## 3 Results and Discussion

### 3.1 Response of the Structure to Variations in *U*

To test how AUO_4_ structures respond to shell-averaged Hubbard *U* corrections, we performed geometry optimizations on *β*-NiUO_4_ in which the values of *U*_Ni–3d_, *U*_U–5f_, and *U*_O–2p_ were varied systematically. This compound was selected for the analysis because it displays the most distorted structure. The results, presented in Figure 2, show a strong and monotonic dependence of both the unit cell volume *V* and the internal distortion parameter *δ*_O_ on the applied *U* values. Specifically, increasing *U*_Ni–3d_ from 0 to 8 eV results in a 4.8% expansion of the volume and a 27% reduction in *δ*_O_ (Figure 2a). Similarly, increasing *U*_U–5f_ from 0 to 8 eV leads to a 3.3% volume expansion and a pronounced reduction in *δ*_O_ by 58% (Figure 2b), which reflects the crucial role of the U-5*f* orbitals for the strongly covalent hybridized axial O1=U=O1 bonds. In contrast, applying *U* corrections to the O–2p shell produces a subtler structural response, as increasing *U*_O–2p_ from 0 to 8 eV entails only a minimal increase in unit cell volume (0.8%) and a reduction in *δ*_O_ by 20% (Figure 2c). The experimental value of *δ*_O_ is best reproduced at *U*_Ni–3d_ ≈ 3 eV, *U*_U–5f_ ≈ 1.5 eV and *U*_O–2p_ ≈ 0 eV, whereas the volume is overestimated for all *U* values, including 0 eV. Qualitatively similar trends are observed for MnUO_4_ and CoUO_4_, suggesting that the *Ibmm* structure of AUO_4_ compounds manifests a high sensitivity to shell-averaged Hubbard *U* corrections; at least when the latter are applied through (ortho-)­atomic projector functions. For ranges of Hubbard *U* values that predict correct electronic band gaps (e.g., *U* > 6 eV for Ni-3d)^51^ the magnitude of the distortions is severely underestimated, especially since the effects of *U*_Ni–3d_, *U*_U–5f_, and *U*_O–2p_ can be expected to stack (albeit not necessarily in a linear way).

**Figure 2:**
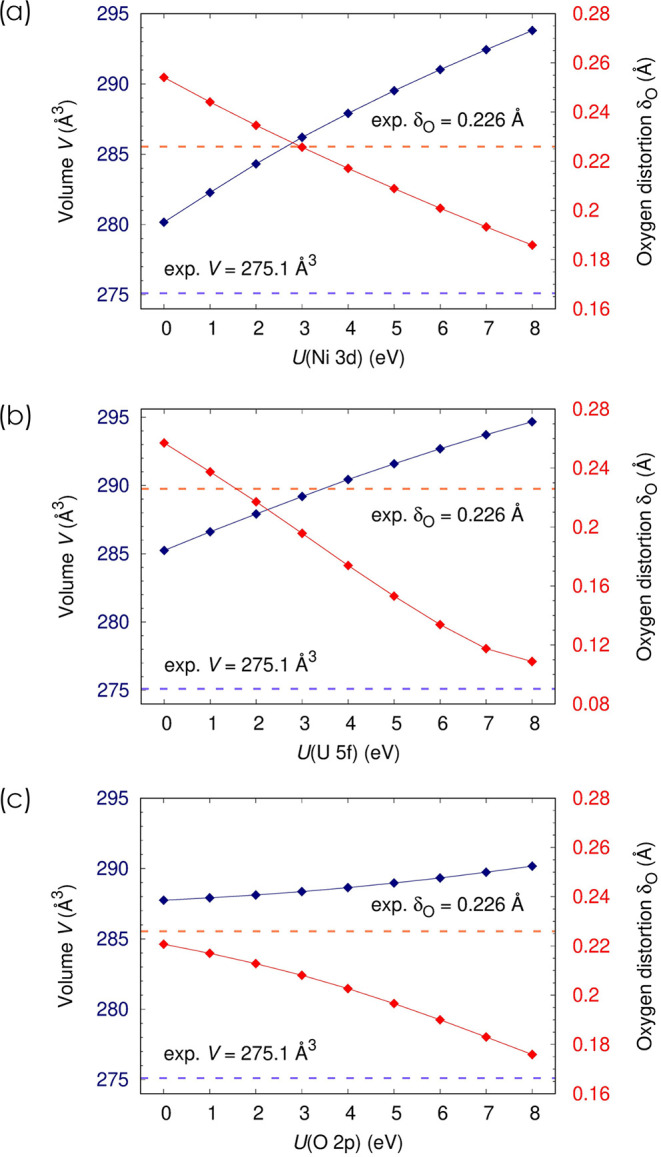
Impact of the Hubbard parameters (a) *U*_Ni–3d_ (at fixed *U*_U–5f_ = 2 eV and *U*_O–2p_ = 1 eV), (b) *U*_U–5f_ (at fixed *U*_Ni–3d_ = 4 eV and *U*_O–2p_ = 1 eV), and (c) *U*_O–2p_ (at fixed *U*_Ni–3d_ = 4 eV and *U*_U–5f_ = 2 eV fixed) on the cell volume and the magnitude of the oxygen distortion parameter in *β*-NiUO_4_. Solid lines serve as a guide for the eye.

Murphy et al.^26^ explained this inconsistent behavior of the *U* correction with “unrealistic” fractional occupation numbers of unoccupied d and f orbitals resulting from the use of atomic orbital projectors.^26^ Due to the functional form of eq 2, fractional occupation numbers induce punitive Hubbard energy contributions that grow quadratically with increasing distance from *λ* = 0 or *λ* = 1 (i.e., *E_U_* is maximized for half-occupied orbitals). Therefore, the minima of the potential energy surface shift toward configurations in which *E_U_* is reduced. For (ortho)-atomic projector functions, this entails the observed volume increase—since larger bond lengths lead to decreased occupations of formally empty orbitals—and causes a linearization of the polyhedral bond angles that reduces the magnitudes of *δ*_U_ and *δ*_O_. In ref 26 these spurious side-effects of *U* corrections were mitigated by using WFs as Hubbard projectors, which yielded occupation numbers much closer to zero or one, or by setting *E_U_* = 0 when using atomic Hubbard projectors. The fact that the problematic contributions to *E_U_* appear to stem from only a few (formally unoccupied) states raises the question of whether there exists an orbital-resolved Hubbard manifold based on (ortho-)­atomic orbital projectors that reduces the sensitivity of the structural parameters to the *U* values and allows for accurate predictions of the structural distortions without resorting to artificial modifications of the energy functional.

### 3.2 Determination of Orbital-Resolved Hubbard Manifolds

To identify such orbital-resolved Hubbard manifolds, we analyze the individual orbitals of the A-3d, U-5f and O-2p shells in terms of overlap and occupation patterns. We emphasize that the consideration of O-2p states for the Hubbard manifold is natural because of their potential frontier-state character and high degree of localization.^52^

#### 3.2.1 Group-Theoretical Analysis of Orbital Splitting

Before turning to practical calculations, we review the ligand-field-induced splitting of the A-3d and U-5f orbitals from a group theory perspective. In the *Ibmm* crystal structure, the A and U cations occupy the 4a and 4e Wyckoff positions, corresponding to *C*_2*h*_ and *C*_2*v*_ site symmetries, respectively. The A-3d orbitals transform as two one-dimensional (nondegenerate) irreducible representations: a *3A*_*g*_ representation with contributions from d_*x* ^2^–*y* ^2^_, d_*z* ^2^_, and d_*xz*_, and a *2B*_*g*_ manifold associated with the d_*xy*_ and d_*yz*_ atomic orbitals. The lobes of d_*xy*_, d_*xz*_ and d_*z* ^2^_ lie between the A–O bond axes and should interact only very weakly with the O-2p orbitals via π overlap (Figure S7). In contrast, the lobes of d_*x* ^2^–*y* ^2^_ and d_*yz*_ run along the equatorial and axial A–O bonds, respectively, giving rise to strong σ overlap. Therefore, despite the significant distortions of the AO_6_ octahedra, the crystal-field splitting pattern of the A-3d shell can be expected to resemble that of a perfect octahedron with *O*_*h*_ site symmetry. We will thus refer to the three lowest-lying orbitals as $\tilde{t_{2g}}$ , and we designate the two higher-energy orbitals as $\tilde{e_{g}}$ , where the tilde denotes the approximate nature of this assignment, since all of the states are nondegenerate in energy.

Moving on, the 5f orbitals of U split into seven nondegenerate levels transforming as *2A* *_1_*, *1A_2_*, *2B_1_*, and *2B_2_.* The *A*_*2*_ representation can be expected to show very little overlap, as the lobes of the corresponding f_*xyz*_ orbital point away from all bond axes (Figure S7). All other representations are linear combinations of two atomic orbitals and exhibit varying degrees of overlap with adjacent O–sp^2^ hybrid orbitals; with the highest energy contributions likely being due to f_*y*(3*x* ^2^–*y* ^2^)_ and f_*xz* ^2^_, whose lobes follow the axial O1=U=O1 and the equatorial O2=U=O2 bond axes, respectively.

#### 3.2.2 Analysis of the Electronic Occupations

Next, we assess the extent of orbital hybridization by carrying out DFT+*U* calculations with shell-averaged trial *U* values and analyze the resulting electronic structures and orbital occupations. For all trial calculations, we employed *U*_U–5f_ = 2.0 eV and *U*_O–2p_ = 1.0 eV. The Hubbard parameters used for the A-site 3d shells were system-specific: *U*_Ni–3d_ = 4.0 eV, *U*_Mn–3d_ = 2.0 eV, and *U*_Co–3d_ = 4.0 eV. These comparatively low trial parameters were chosen to avoid analyzing an already overcorrected electronic structure, as spurious effects due to shell-averaged Hubbard *U* corrections often set in at *U* > 4 eV.^19,53^ The parameter *U*_Mn–3d_ was assigned a smaller value than *U*_Ni–3d_ and *U*_Co–3d_ because previous studies indicate that the Mn-3d shell is often less affected by SIEs than Ni-3d and Co-3d.^51^

The eigenvalues of the occupation matrix, presented in Table 1, clearly reflect the crystal-field splitting between the $\tilde{e_{g}}$ and $\tilde{t_{2g}}$ states of Ni in *β*-NiUO_4_. While eigenstates ν_3_ to ν_5_ are fully occupied (*λ* > 0.98) in both spin channels, *λ*_1_^↓^ and *λ*_2_^↓^ are significantly closer to zero (albeit still well above 0.1), indicating formally empty ( $\tilde{e_{g}}$ ) states. The remaining eigenstates ν_3_^↓^ to ν_5_^↓^ are again fully occupied, which is consistent with the minority-spin $\tilde{t_{2g}}$ states of a high-spin d^8^ configuration. This assignment is confirmed by the PDOS shown in Figure 3a, where it can be recognized that the Ni contributions to the conduction bands are exclusively composed of $\tilde{e_{g}}$ -like ν_1_^↓^ and ν_2_^↓^ states (Figure 3e). The occupancies of the 3d orbitals of Mn^2+^ and Co^2+^ in MnUO_4_ and CoUO_4_ show a qualitatively very similar trend (Table S1), and are in line with the expected respective d^5^ and d^7^ high-spin configurations that were also found by Murphy et al.^26^ In all of the three materials, the 3d shells host formally empty yet significantly occupied $\tilde{e_{g}}$ -like eigenstates in the spin-minority manifold that are clearly distinguishable from other empty states by virtue of their elevated eigenvalues (*λ*> 0.1), in the PDOSs (Figures S1 and S2) and when visualized in real space (Figure S8). While it is generally desirable to correct all states contributing to the frontier orbitals, here, we chose to exclude the $\tilde{e_{g}}$ -like eigenstates from the Hubbard manifold because their elevated occupation eigenvalues (stemming from hybridization between $A‐\tilde{e_{g}}$ and ligand p orbitals^54^) give rise to spurious energy and force contributions.^19,32^ We emphasize that this can be seen as an *ad hoc* solution to the unsatisfactory projectability of the $\tilde{e_{g}}$ manifold when atomic-like projectors are employed. Another solution (which we do not follow here) is to switch to a Wannier function projector basis, as practiced in ref 26 (see the SI therein for a comparison of WF vs NAO occupation numbers). A possible orbital-resolved manifold for the 3d shells therefore consists of the $\tilde{t_{2g}}$ states, which are reasonably represented by the (ortho-)­atomic projector orbitals and thus exhibit occupation eigenvalues close to either 0 or 1. This target subspace includes ν_3_–ν_5_ for *β*-NiUO_4_ (both spin channels), ν_3_^↑^–ν_5_^↑^ and ν_1_^↓^–ν_3_^↓^ for MnUO_4_, and ν_3_^↑^–ν_5_^↑^, ν_1_^↓^, as well as ν_4_^↓^–ν_5_^↓^ for CoUO_4_. The assignment of the spin-majority eigenstates to either $\tilde{t_{2g}}$ or $\tilde{e_{g}}$ must rely on the eigenvectors, as the corresponding eigenvalues are all close to unity and thus do not allow for a distinction between the two manifolds. Despite their numerically full occupation, the spin-majority $\tilde{e_{g}}$ -like eigenstates were also removed from the Hubbard manifold because fully occupied states are still affected by the Hubbard correction via the potential shift of −*U*/2 resulting from eq 3. Moreover, an imbalanced correction of spin orbitals (i.e., correcting more spin-majority than spin-minority orbitals) would emulate a Hund *J* term,^55,56^ which might be useful for addressing fractional spin errors, but a systematic treatment of such effects would go beyond the scope of this study.

**Table 1:** Eigenvalues *λ*_*i*_^*I*^ Corresponding to Respective Eigenstates ν_*i*_^*I*^ in *β*-NiUO_4_, Obtained from a DFT+*U* Calculation with Trial Hubbard Parameters *U*_Ni–3d_ = 4.0 eV, *U*_U–5f_ = 2.0 eV and *U*_O–2p_ = 1.0 eV.

		eigenvalue						
atom	spin	*λ*_1_	*λ*_2_	*λ*_3_	*λ*_4_	*λ*_5_	*λ*_6_	*λ*_7_
U	↑	0.063	0.107	0.175	0.196	0.247	0.285	0.417
	↓	0.062	0.108	0.150	0.165	0.202	0.241	0.378
Ni	↑	0.972	0.989	0.992	0.994	0.996	–	–
	↓	0.164	0.221	0.983	0.987	0.991	–	–
O_1_	↑	0.748	0.765	0.812	–	–	–	–
	↓	0.736	0.766	0.810	–	–	–	–
O_2_	↑	0.744	0.799	0.802	–	–	–	–
	↓	0.733	0.759	0.799	–	–	–	–

**Figure 3:**
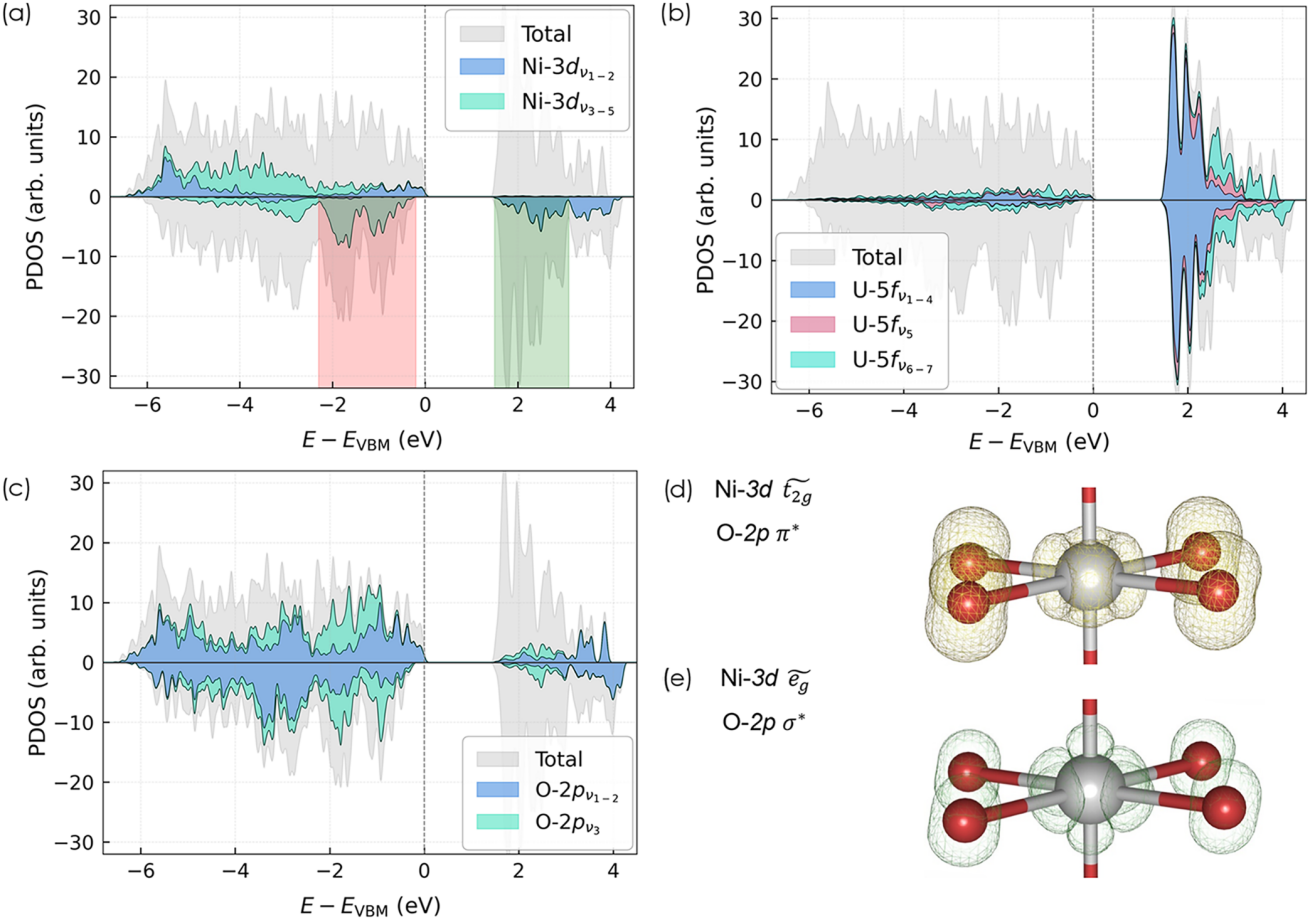
Stacked PDOS for (a) Ni-3d, (b) U-5f, and (c) O-2p orbitals in *β*-NiUO_4_, obtained from trial DFT+*U* calculations. Panels (d) and (e) show the integrated local density of states for the Ni-3d energy intervals indicated by red and green shading in panel (a) at isovalues 0.0184 and 0.0079 *e* ^–^·Å^–3^, respectively.

For uranium, the PDOS indicates that most 5f contributions to the electronic structure are located in the conduction band (Figure 3b); however, the projected occupations of some eigenstates are significantly larger than zero despite the formal 5f^0^ configuration of U^6+^. Particularly, the high-energy eigenstates ν_5_, ν_6_ and ν_7_ exhibit eigenvalues above 0.2 (in both spin channels), with *λ*_7_ growing as large as 0.45 in MnUO_4_ (Tables 1 and S1). Given the continuous nature of the U-5f occupation spectrum, the definition of an orbital-resolved Hubbard manifold is somewhat ambiguous and cannot be guided by symmetry considerations alone. Based on their comparatively low occupation eigenvalues, we considered the four least-occupied eigenstates (ν_1_–ν_4_) to be sufficiently localized to be included in the Hubbard manifold, whereas ν_5_–ν_7_ were not targeted by Hubbard corrections to avoid overcorrection due to heavy ligand admixture. To evaluate the robustness of our choice, we examined an alternative definition of the target subspace in which ν_5_ was also included. This variation led to only minor changes in the computed values of the *U* parameters (Table S2).

While partially filled d and f shells are typically the focus of Hubbard *U* corrections, the possibility that O-2p states might also be localized^52^ (and thus require correction of SIEs) is often overlooked. In the present ternary monouranates, the (distorted) trigonal planar coordination geometries of both O sites, with bond angles between 105° and 140°, suggest the presence of three sp^2^ hybrid orbitals mediating the interactions between the oxygen atom and the metal centers, and one lone-pair orbital. The sp^2^ hybrid orbitals display significant σ-overlap with neighboring A- and U-site cations (for example, Figure 3e shows a σ_d–p_^*^ orbital forming part of the lowest conduction bands), while the unhybridized p_*x*_/p_*y*_ orbitals (depending on whether the site is O1 or O2) interact only slightly with the neighboring sites via π overlap. The occupation eigenvalues of O-2p are far from one, ranging from *λ* ≈ 0.73 to 0.82 (Table 1 for β-NiUO_4_, Table S1 for MnUO_4_ and CoUO_4_). However, there is a noticeable gap between the numerical values of *λ*_2_ (≈ 0.76) and *λ*_3_ (≈ 0.80), except for the spin-up manifold of O2 where both values are similar. Careful analysis of the PDOS shows that energy ranges where $A‐\tilde{t_{2g}}$ states dominate the electronic structure (e.g., the red shaded area in Figure 3a) also show large contributions due to ν_3_ of O-2p. By visualizing the eigenstates of the occupation matrix (Figure S8), it becomes evident that ν_3_ corresponds to the nominally unhybridized p_*x*_(p_*y*_) orbital of O1 (O2). We argue that the orbital-resolved Hubbard manifold of O should comprise only this eigenstate, which exhibits weaker hybridization and a more atomic-like character. In contrast, the sp^2^-hybridized states are improperly represented by (linear combinations of) 2p OAOs (Figure S7), potentially rendering a correction of SIEs through Hubbard *U* terms difficult. This assessment is further backed by the orbital-energy diagram of the NiO_6_ octahedron depicted in Figure 4, where it can be seen that a large number of symmetry-adapted linear combinations (SALCs) remain purely oxygenic. These *ungerade* states (e.g., 6*A*_u_, 9*B*_u_) account for most of the levels just below the valence-band maximum, are nonbonding to weakly antibonding (Figure S9), and indeed show a strong atomic-like p orbital character. On the other hand, the O-2p contributions to the *11A_g_* frontier orbital, which is an antibonding d–pσ* molecular orbital (MO), display heavy admixture with Ni-3d orbitals, highlighting the difficulty of representing these states with a conventional (ortho-)­atomic projector basis. For this reason ν_1_ and ν_2_ were not included in the orbital-resolved Hubbard manifold of O-2p, whereas ν_3_ was.

**Figure 4:**
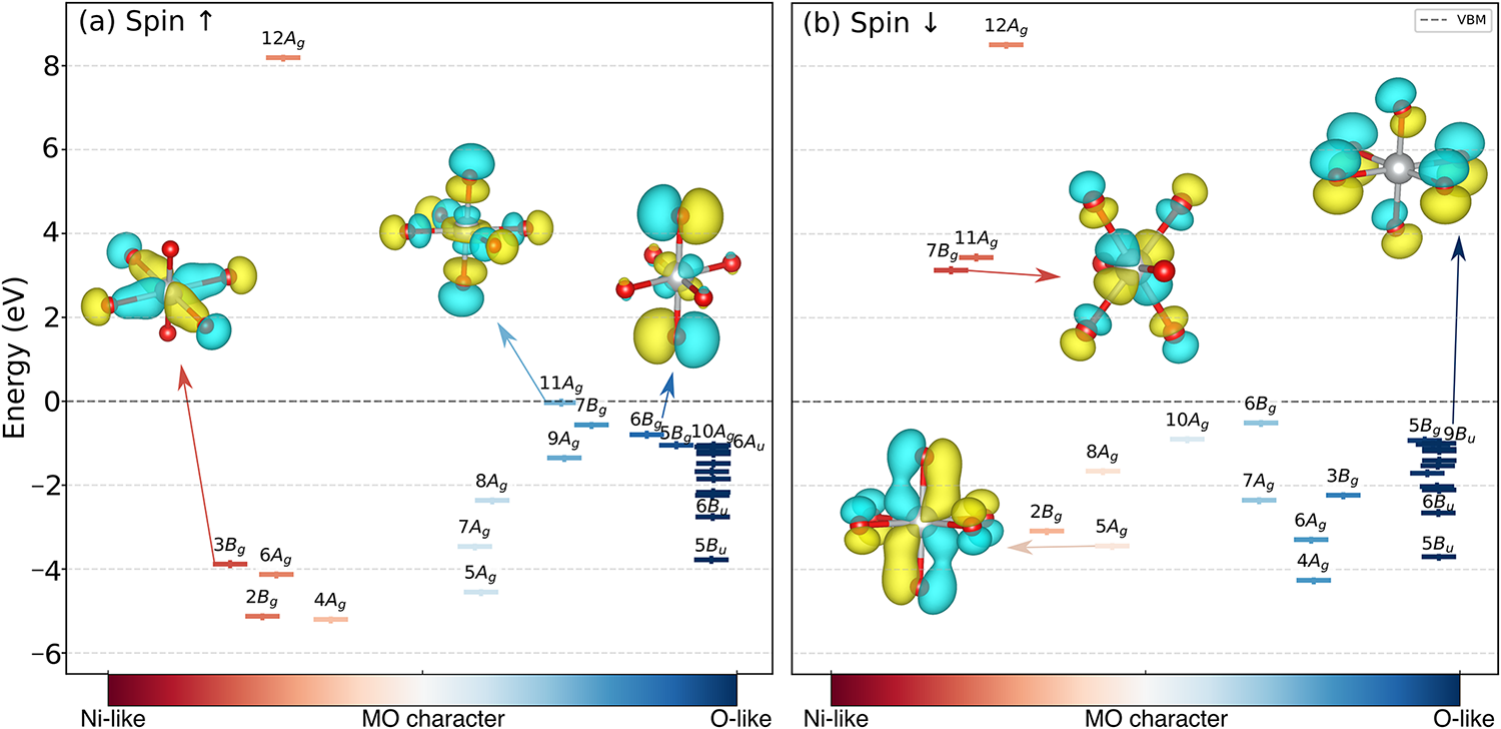
Molecular orbital (MO) diagram of an NiO_6_ octahedron in NiUO_4_ indicating the relative TM–3d vs O-2p character of the MOs on the *x*-axis. The insets show the MOs corresponding to a few irreducible representations of interest. Data was generated by applying the linear combination of fragment orbitals method^41^ implemented in LOBSTER^42^ to the ground state of the trial DFT+*U*(OAO) setup.

### 3.3 Evaluation of the Hubbard *U* Parameters

Having established orbital-resolved Hubbard manifolds for all frontier shells, we proceed to evaluate the respective Hubbard parameters from LR-cDFT and compare the outcome for different choices of Hubbard manifolds. In the traditional shell-averaged manifold, Hubbard corrections are applied to all A-3d, U-5f, and O-2p orbitals. The OR-DFT+*U* approach systematically refines this definition of the target subspace through the following sequence of setups with increasingly selective manifolds: (1) The U-5f subspace is restricted to eigenstates ν_1_–ν_4_, while shell-averaged *U* corrections are retained for the A-site 3d and O-2p states; (2) as in (1), but with an orbital-resolved *U* applied to A-site $\tilde{t_{2g}}$ states; (3) as in (2), but additionally limiting the O-2p correction to the p_*x*_ (O1) and p_*y*_ (O2) states.

Table 2 shows the *U* values obtained for all choices of Hubbard manifolds and for each compound. While still falling in typical ranges,^48,52^ all shell-averaged *U* values are comparatively large, and even exceed those reported by Murphy et al.^26^ by 1 to 3 eV. The reason for this discrepancy is likely rooted in the use of different Hubbard projector functions: while Murphy et al.^26^ employed nonorthogonalized atomic orbitals, here, OAO were used.

**Table 2:** Self-Consistent Hubbard *U* Parameters Obtained From LR-cDFT for Four Setups (One Shell-Averaged Plus Three Orbital-Resolved) with Differently Defined Hubbard Manifolds^a^

system		*U* (eV)		
Shell-Averaged	A-3d	U-5f	O1–2p	O2–2p
*β*-NiUO_4_	7.03 (6.6)	3.65 (2.6)	7.56	7.97
MnUO_4_	7.34 (4.4)	3.92 (2.7)	7.45	7.82
CoUO_4_	6.19 (5.2)	3.77 (2.7)	7.54	7.94
Setup (1)	A-3d	U-5f_ν1−ν_4__	O1–2p	O2–2p
β-NiUO_4_	7.03	1.37	7.59	7.97
MnUO_4_	7.17	1.38	7.49	7.87
CoUO_4_	6.20	1.36	7.56	7.94
Setup (2)	$A‐\tilde{t_{2g}}$	U-5f_ν1−ν_4__	O1–2p	O2–2p
β-NiUO_4_	7.40	1.33	7.50	7.78
MnUO_4_	1.27	0.93	7.40	7.74
CoUO_4_	2.33	1.22	7.47	7.77
Setup (3)	$A‐\tilde{t_{2g}}$	U-5f_ν1−ν_4__	O1–2p_x_	O2–2p_y_
β-NiUO_4_	7.68	1.26	4.14	4.13
MnUO_4_	1.46	1.01	4.25	4.18
CoUO_4_	2.56	1.15	4.11	4.14

a The values in parentheses are those derived and applied by Murphy et al.^26^

Moving forward to setup (1), excluding the most hybridized eigenstates ν_5_–ν_7_ from the Hubbard manifold of U-5f prompts a strong and consistent reduction in the corresponding on-site interaction parameters of all three isomorphous compounds, from *U*_U–5f_ ≈ 3.8 eV to *U*_U–5*f*ν_1_–ν_4__ ≈ 1.4 eV. At the same time, the A-3d and O-2p manifolds barely respond to the exclusion of ν_5_–ν_7_, maintaining the same *U* values as in the shell-averaged manifold.

A more drastic drop is observed in setup (2) for MnUO_4_ and CoUO_4_, where substituting the shell-averaged *U*_A–3d_ correction by a $\tilde{t_{2g}}$ -specific one leads to *U* parameters as low as ≈1.3 eV (MnUO_4_) and ≈2.3 eV (CoUO_4_), respectively. The 1 eV difference between $U_{\mathrm{Mn}-\tilde{t_{2g}}}$ and $U_{\mathrm{Co}-\tilde{t_{2g}}}$ can be understood by recognizing that the $\tilde{t_{2g}}$ subspace of Mn contains three electrons [ ${\tilde{t_{2g}}}^{3}\left(↑\right)$ ], whereas that of Co holds five [ ${\tilde{t_{2g}}}^{3}\left(↑\right)$ and ${\tilde{t_{2g}}}^{2}\left(↓\right)$ ]. In *β*-NiUO_4_, however, the $\tilde{t_{2g}}$ subspace is fully occupied [ ${\tilde{t_{2g}}}^{3}\left(↑\right)$ and ${\tilde{t_{2g}}}^{3}\left(↓\right)$ ], which causes the manifold to be quite insensitive to perturbations, i.e., its occupations barely change during the course of the LR-cDFT calculations.^57^ This results in a small numerical response that ultimately translates into a large orbital-resolved Hubbard parameter of $U_{\mathrm{Ni}-\tilde{t_{2g}}}=7.4$ eV. Interestingly, the transition to orbitally resolved A-3d manifolds also affects the *U* values of the other manifolds, particularly in MnUO_4_, where *U*_U–5f_ν1_–ν_4__ drops by 0.4 eV compared to setup (1), whereas *U*_O1–2p_ and *U*_O2–2p_ decrease only slightly by ≈0.1 eV. This demonstrates the importance of the $\tilde{e_{g}}$ states for both intrashell screening ( $\tilde{t_{2g}}\Leftrightarrow \tilde{e_{g}}$ ) and intersite interactions between the A-3d shell and adjacent ligand 2p orbitals.

Finally, with setup (3) the *U* values of oxygen also experience a strong reduction, as the Hubbard *U* correction to O-2p is restricted to the localized p_*x*_ (O1) and p_*y*_ (O2) orbitals, respectively. Remarkable is not only the drop in values by itself, from ≥7.4 down to ≈4.2 eV, but also the tightening of the spread (i.e., the difference between the largest and the smallest *U*_O–2p_ value), which amounts to ≈0.5 eV in the previous setups but shrinks to only 0.1 eV in setup (3). The fact that *U*_O–2p_x/*y*__ is essentially constant, regardless of the A-site cation or the crystallographic site, is a testimony to the almost nonbonding (lone-pair) nature of these orbitals. Nevertheless, with *U* values on the order of 4.2 eV, they severely contribute to deviations from PWL; and intriguingly do so much more than the $A‐\tilde{t_{2g}}$ and U–5f_ν_1_–ν4_ subspaces.

The strong reduction in *U* parameters upon transition to orbital-resolved manifolds highlights an important feature of the linear-response formalism, namely that only screening channels outside the target subspace contribute to the effective interaction strength.^19,56,58^ When perturbing entire 5f, 3d, or 2p shells, important interactions (particularly intrashell ones like $\tilde{t_{2g}}\Leftrightarrow \tilde{e_{g}}$ ) are prevented from screening the perturbation, which leads to large *U* values due to small (apparent) responses. This problem is aggravated by the use of OAO projectors, which often misattribute ligand electrons (e.g., belonging to O-sp^2^ hybrid orbitals) to metal orbitals, thus suppressing intershell screening pathways. By instead targeting only the most localized frontier orbitals, one mitigates both issues: the relevant intrashell screening channels remain active, and the misattribution of ligand electrons remains unpunished, as the $\tilde{e_{g}}$ and U-5f_ν5−ν7_ manifolds are not included in the Hubbard manifold. For the AUO_4_ systems investigated here, the orbitally resolved interaction parameters are therefore expected to be more representative of the physical reality than their shell-averaged counterparts.

### 3.4 Structural Distortions

To assess the impact of differently defined Hubbard manifolds on crystal structure predictions, we evaluate the key distortion parameters θ, δ_U_ and δ_O_ defined in Section 2.2. These distortions are highly sensitive to the underlying electronic structure (and vice versa), as illustrated by the PDOSs obtained using different DFT­(+*U*) schemes in Figures S3–S5 of the SI, and thus provide a meaningful benchmark for this purpose. The PDOSs computed with the shell-averaged DFT+*U* method [(b) panels] bear some similarity with those of DFT+*U*(WF) [(f) panels]; however, the latter predicts a valence band edge of predominant A-3d character for MnUO_4_ and CoUO_4_, whereas in the former, these band edges are strongly dominated by O-2p states. The gradual exclusion of the hybridized states from the Hubbard manifold [(c), (d), and (e) panels] leads to a PDOS more closely resembling that of PBEsol [(a) panels], with the A-3d dominated states located close to the valence band maximum. Regarding the band gaps, DFT+*U*(WF), shell-averaged DFT+*U* method and hybrid functional [HSE06,^59^ provided here as a reference (Figure S6)] consistently yield gaps on the order of ≈2 eV, whereas smaller gaps <1 eV are obtained for the OR-DFT+*U* setups (2) and (3), except for MnUO_4_, which is predicted to be metallic. Moreover, the OR-DFT+*U* setups (2) and (3) and HSE06 predict broader valence and conduction bands than DFT+*U*(WF) and shell-averaged DFT+*U*, whose bands are comparatively denser. Given the lack of experimental data on the electronic structure of these materials, no definitive conclusion can be drawn regarding the spectral accuracy of the various methods. Nevertheless, although DFT+*U*(WF) qualitatively gives the best match to the HSE06 results, OR-DFT+*U* setups (2) and (3) show better agreement in terms of band widths. These subtle similarities and differences are taken into account when discussing the accuracy of the different methods in predicting the structural distortions.

Figure 5 presents the results for uncorrected DFT (PBEsol), the shell-averaged manifold (shell-averaged DFT+*U*) and the three orbital-resolved Hubbard manifolds defined above, applied using the parameters reported in Table 2. Also shown for comparison is the DFT+*U*(WF) data taken from in ref 26 (details of these calculations are provided in Section IA of the SI). Importantly, the projector basis (OAO) is identical for the shell-averaged calculations and the orbitally resolved ones, so that differences between these approaches arise solely from the specification of the target subspace rather than from the projectors.

**Figure 5:**
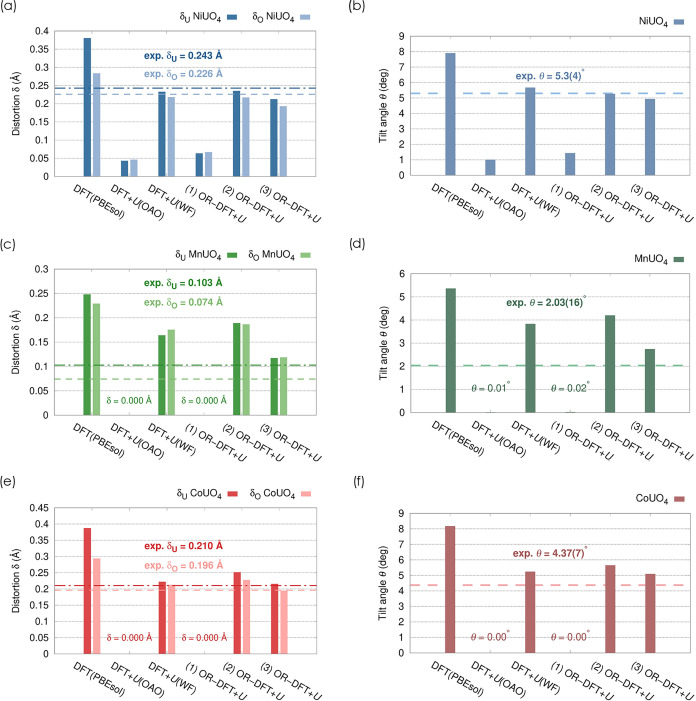
Off-center displacements of uranium, *δ*_U_, and O2 atoms, *δ*_O_ (a, c, e), and octahedral tilt angle *θ* in (b, d, f) as obtained from bare PBEsol and DFT+*U* calculations using differently defined Hubbard manifolds (see text and Table 2). The DFT+*U*(WF) data points were taken from ref 26.

The uncorrected PBEsol functional significantly overestimates the lattice distortions in all three compounds. The most pronounced case is MnUO_4_ (Figure 5c,d), where the predicted values of *δ*_U_ and *θ* massively exceed the experimental estimates,^26^ by 241% and 264%, respectively. For β-NiUO_4_ and CoUO_4_ (Figure 5a,b and e,f, respectively), the deviations are slightly less dramatic but remain significant; for instance, *θ* is off by nearly 90% in CoUO_4_.

If these overestimations stem from SIEs (and the associated spurious overdelocalization of charge), Hubbard *U* corrections should rectify or at least mitigate them, provided that the Hubbard manifold is defined such that (only) localized frontier states are targeted by the correction.^10,19^ This is seemingly not the case for shell-averaged DFT+*U*, which suppresses structural distortions across all compounds. While in *β*-NiUO_4_, *δ*_U_, *δ*_O_, and *θ* are severely underestimated by up to 500%, MnUO_4_ and CoUO_4_ are even entirely transformed into the higher-symmetry *Cmmm* configurations, as all of of their distortion parameters drop to zero (barring numerical noise). The DFT+*U*(WF) scheme represents a notable improvement with respect to both uncorrected DFT and shell-averaged DFT+*U*, as the Wannier function basis adequately represents frontier orbitals that preserve spatial localization while minimizing overlap with ligand states. With the improved WF Hubbard projectors, the distortion parameters calculated for *β*-NiUO_4_ and CoUO_4_ are found in good agreement with the experiment, deviating by less than 20%. Only for MnUO_4_ the accuracy is less satisfactory, as *δ*_U_, *θ* and *δ*_O_ remain overestimated by 59%, 89% and 136%, respectively. The reasons for this inconsistency will be discussed later.

OR-DFT+*U* with setup (1) offers no improvement over shell-averaged DFT+*U*, as the structural distortions remain suppressed almost entirely across all compounds. This is in spite of the considerable difference between the numerical values of *U*_U–5fν_1_–ν_4__ and *U*_U–5f_. However, the Hubbard corrections to U–5f mainly affect states in the conduction band, which clearly do not influence the ground-state ionic structures. This result supports previous findings indicating that the symmetry of ternary monouranates is mainly controlled by the A-site cations.^26,49^

Hence, expanding the orbital resolution to the A-sites’ $\tilde{t_{2g}}$ orbitals within setup (2) markedly improves the accuracy of predictions for *β*-NiUO_4_ and CoUO_4_, with *δ*_U_, *δ*_O_ and *θ* closely matching both experimental values and the results of DFT+*U*(WF). However, and similar to DFT+*U*(WF), setup (2) still overestimates the distortions in MnUO_4_. It is worth noting that Mn^2+^ ions are comparatively large, with an ionic radius of 0.83 Å that exceeds the radii of both Co^2+^ (0.745 Å) and Ni^2+^ (0.69 Å).^60^ Consequently, Mn^2+^ shows a stronger tendency to hybridize with neighboring O-2p states that is also reflected in the more pronounced variations of the Hubbard *U* values (Table 2). In view of this, it is no surprise that setup (3), where the Hubbard manifold of O is restricted to the localized p_*x*_/p_*y*_ orbitals (in addition to orbitally resolved corrections to U-5f and A-3d), cures the systematic overestimation of distortion parameters in MnUO_4_. Fortunately, the orbital-resolved treatment of O-2p affects the already good predictions of the other two compounds to a much lesser degree: minor improvements result for CoUO_4_, where *δ*_U_ and *δ*_O_ match the experimental value almost exactly, whereas the accuracy is minimally deteriorated for *β*-NiUO_4_. Therefore, setup (3) provides the most consistent overall accuracy, delivering very good predictions for all distortion parameters and across all compounds. Its dramatic edge over setup (2) and DFT+*U*(WF) in MnUO_4_ shows the importance of avoiding the correction of strongly hybridized states, here consisting in the bonding σ-states that form between A- $\tilde{e_{g}}$ and the O-sp^2^ hybrid orbitals. More in general, the improvements achieved due to setups (2) and (3) demonstrate that a physically meaningful use of Hubbard *U* corrections requires careful disentanglement of localized and delocalized states. This is particularly crucial for systems where orbital hybridization plays an important role. Additional relative energies of the *Cmmm* and *Ibmm* phases (Table S4) corroborate the structural analysis: setups (2) and (3) yield the correct ground-state ordering for *β*-NiUO_4_ and CoUO_4_, and the residual MnUO_4_ deviation vanishes after removing the Hubbard contribution, consistent with ref 26.

### 3.5 Projector Mismatch as a Source of Spurious Hubbard Forces

The profound impact of the Hubbard manifold on the accuracy of structural predictions for AUO_4_ compounds demands clarification. While it is generally known that the ionic ground state of DFT+*U* can differ significantly from that of the bare functional, it is not understood why and under which circumstances DFT+*U* leads to oversymmetrization of atomistic structures, as observed here and reported elsewhere for diverse TM oxides.^26,53,61–63^ In the following, we try to identify the root cause of these deviations and demonstrate why certain Hubbard projector functions or target manifolds perform better than others.

It follows from the Hellmann–Feynmann theorem that the Hubbard energy functional (c.f. eq 1) contributes an additional term **F**_*U*_^*I*^ to the total force acting on an ion *I* ^44,64^

6 $$F_{U}^{I}=-\frac{\partial E_{U}}{\partial R^{I}}=-\sum_{k,v,\sigma }\left\langle \psi_{k,v}^{\sigma }\left|\frac{\partial V_{U}^{\sigma }}{\partial R^{I}}\right|\psi_{k,v}^{\sigma }\right\rangle$$

where **R**_*I*_ denotes the position of the *I*th atom. Eq 6 implies that a finite Hubbard force arises whenever the derivative $\frac{\partial V_{U}^{\sigma }}{\partial R^{I}}$ is nonzero. Recalling eq 3 and assuming a negligible dependence of *U* on the position, this condition is fulfilled if a displacement of ion *I* (∂**R**_*I*_) modifies the occupation of an eigenstate (∂*λ*_*i*_^*Iσ*^), for instance due to an increase or decrease in its overlap with occupied KS states. Note that $\frac{\partial V_{U}^{\sigma }}{\partial R^{I}}$ can also be nonzero if the projectors themselves vary, e.g., due to reorthogonalization of OAO projectors. In the following, however, we neglect these so-called Pulay forces^44^ and focus instead on the electronic “density response”.^65^ Because of the form of the Hubbard energy functional, eigenstates with occupation *λ*_*i*_^*I*σ^ < 0.5 will experience a force that tends to further reduce their occupation (driving *λ*_*i*_^*I*σ^ → 0) by lowering their overlap with occupied KS wave functions. Conversely, eigenstates with *λ*_*i*_^*I*σ^ > 0.5 will tend to increase their occupation. The central question is: why would an eigenstate exhibit an occupation far from both zero and one to begin with? This consideration allows us to distinguish two fundamentally different scenarios under which nonzero Hubbard forces arise:

In scenario (i), $\frac{\partial \lambda_{i}^{I\sigma }}{\partial R^{I}}\neq 0$ because the occupation of an eigenstate deviates from zero or one due to violations of the PWL condition. Hence, the resulting forces act to counter the artificial delocalization of states caused by SIEs. In scenario (ii), $\frac{\partial \lambda_{i}^{I\sigma }}{\partial R^{I}}\neq 0$ because the (localized) Hubbard projector functions do not accurately represent the true electron distribution in the system and produce occupation numbers that lie between zero and one, independently of the presence of SIEs. Here, the resulting forces bias the charge density distribution *ρ*(**r**) (and therefore also the ionic structure) toward alignment with the Hubbard projector functions. Such forces are artificial and unphysical, since there is no fundamental reason why the electron density of a system should conform to that of an arbitrarily defined set of projector functions. Popular atomic-like projector functions, for example (including NAO, OAO, PAW, and LMTO projectors), are constructed based on radial Schrödinger equations for isolated atoms in their neutral charge state. However, the isolated-atom model may not be a reliable approximation for real compounds. This is especially the case for materials with strong covalent bonding (where the quantum numbers *n*, *l* and *m* can lose their physical meaning), for structures with highly distorted bond angles, or when the radius of a charged ion deviates significantly from that of the neutral atom.

Figure 6 illustrates this issue by showing the mismatch between the unoccupied (spin-down) $\tilde{e_{g}}$ -like KS orbitals around the Ni^2+^ ion in *β*-NiUO_4_ and the diagonal projector orbitals $\phi_{\tilde{e_{g}}}^{↓}\left(r\right)$ corresponding to eigenstates ν_1_^↓^ and ν_2_^↓^ of the occupation matrix. These eigenstates, which are linear combinations of the OAO projectors (Figures S7 and S8), are indeed fractionally occupied (*λ*_1_^↓^ ≈ 0.16, *λ*_2_^↓^ ≈ 0.22, c.f. Table 1). Therefore, applying Hubbard *U* corrections to this manifold induces an unphysical symmetrization of the structure, as the minimum of this DFT+*U* ground state shifts to a configuration where the overlap of the occupied KS states with ν_1_ and ν_2_ is minimized. Note that such artifacts often remain negligible in highly symmetric systems, including the well-investigated solids FeO and NiO. These systems exhibit coordination polyhedra with ideal *O*_*h*_ site symmetries, where the angular shape of atomic projectors (e.g., 90° or 180° for d orbitals) coincides with the metal–ligand bond axes.

**Figure 6:**
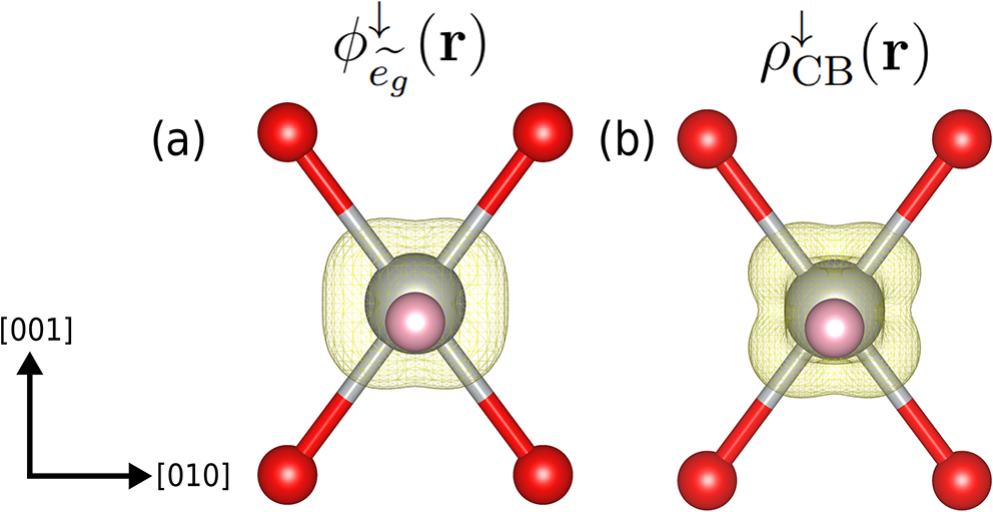
Comparison for *β*-NiUO_4_ between the spatial extension of (a) the squared modulus of the Hubbard projector orbitals, $|\phi_{\overset{\sim }{e_{g}\;}}^{↓}\left(r\right){|}^{2}\equiv |\phi_{1}^{↓}\left(r\right)+\phi_{2}^{↓}\left(r\right){|}^{2}=|\overset{5}{\underset{m=1}{\sum }}\left(\overset{2}{\underset{i=1}{\sum }}\nu_{\mathrm{mi}}^{↓}\right)\varphi_{m}\left(r\right){|}^{2}$ , representing the formally unoccupied $\tilde{e_{g}}$ -like orbitals, and (b) the (pseudo)-charge density due to the KS wave functions of the lowest conduction bands in the spin ↓channel in the range from 1.8 to 4.2 eV w.r.t. valence band maximum, ρ_CB_^↓^(**r**) = ∑_v∈CB_∑_**k**_ |ψ_**k**,*v*_^↓^(**r**)|^2^ [see Figure 3(a)]. It is the mismatch between projectors and KS wave functions that gives rise to the fractional occupation numbers *λ*_1_^↓^ and *λ*_2_^↓^. The color code is the same as in Figure 1.

The observation of an artificial force contribution provides a rationale for the improvements observed when employing either WF projectors or OR Hubbard manifolds. In the former case, it is the projector functions themselves that adapt to the charge density, thus resulting in a less fractional eigenvalue spectrum that yields no spurious force contributions. Conversely, in the latter case, the inadequate eigenstates are simply excluded from the correction by setting *U*_*i*_ = 0 for all respective *i*. Note that spurious Hubbard forces due to projector mismatch can also entail “under-symmetrization”, as demonstrated by the improvements in the structural predictions for MnUO_4_ upon switching from OR manifold (2) to manifold (3). In manifold (2), the correction targets three sp^2^ hybrid orbitals (with bond angles of approximately 120°) plus an unhybridized nonbonding state using three (ortho-)­atomic 2p projectors (p_*x*_, p_*y*_, p_*z*_). While the nonbonding state (eigenstate ν_3_) is well represented by one of the projectors, it is evident that the remaining two projectors (or any linear combination thereof) cannot reproduce the spatial extent of the three sp^2^ hybrid orbitals. Consequently, correcting eigenstates ν_1_ and ν_2_ of the O-2p shell drives the trigonal planar geometry toward an atomic-like one, in which the bond angles are closer to 90°. In the AUO_4_ structures considered here, such distortions increase the deviation from the more symmetric *Cmmm* geometry, most significantly in MnUO_4_, where interactions between the A-site and neighboring O atoms are strongest due to the relatively large radius of Mn^2+^.

## 4 Concluding Remarks

Using an orbital-resolved DFT+*U* scheme,^19^ we have demonstrated how the choice of the Hubbard manifold affects the ground-state electronic and structural properties of MnUO_4_, CoUO_4_ and *β*-NiUO_4_. With (ortho-)­atomic projector functions, the characteristic distortions of these monouranates are accurately reproduced only when the Hubbard manifold is restricted to the most localized subset of orbitals within the A-3d, U-5f, and O-2p shells. In contrast, applying Hubbard *U* corrections to more delocalized states within these shells markedly reduces the accuracy. An exception is found for the U-5f shell, where the structural distortions are insensitive to the choice of Hubbard manifold, although changes in the electronic structure are still observed (see Figures S3–S5). The excessive sensitivity of the ionic structure to the choice of the Hubbard manifold stems from an implicit, artificial contribution in the Hubbard force expression, which aligns the computed charge density *ρ*(**r**) (and thus, the ionic positions) with the diagonalized (ortho-)­atomic projectors ϕ_*i*_^*I*σ^(**r**).

Two strategies can help mitigate these artifacts: either one employs Hubbard projector functions that reflect the local bonding environment more faithfully—such as Wannier functions (as practiced in ref 26.)—or one excludes strongly hybridized states (where the mismatch between *ρ*(**r**) and ϕ_*i*_^*I*σ^(**r**) is large) from the Hubbard manifold using OR-DFT+*U*, as done here. The success of both approaches, DFT+*U*(WF) and OR-DFT+*U*, hinges on an appropriate choice of the Hubbard manifold (in fact, this applies to all DFT+*U* approaches, including the shell-averaged one). Within the DFT+*U*(WF) framework, this involves the selection of energy windows and sometimes also the disentanglement of bands; for OR-DFT+*U* with atomic-like Hubbard projectors, one must carefully determine which eigenstates of the occupation matrix exhibit true on-site character, e.g., by analyzing the eigenvalue spectrum. These steps typically require chemical intuition and some degree of trial-and-error, although recent progress on the automation of the Wannierzation step^66,67^ has significantly reduced the effort associated with using the DFT+*U*(WF) formalism. DFT+*U*(WF) might outperform OR-DFT+*U* in structures where the mismatch between the true charge density and the shape of atomic-like projectors is extreme, or where Hubbard corrections need to be applied to electronic states localized between atoms (cf. intersite terms of refs 63,68 ). This can be important when atomic-like projector functions cannot adequately describe a localized manifold, for instance highly localized sp^3^ hybrid orbitals. A WF-based approach could still work in such cases because Wannier functions do not make any assumptions regarding the spatial extension of the localized states. On the other hand, owing to its conceptual simplicity, OR-DFT+*U* can leverage existing routines for the calculation of forces and stresses, thereby enabling structural relaxations and *ab initio* molecular dynamics, which are often not feasible or not yet available within existing DFT+*U*(WF) implementations. Furthermore, due to a more realistic representation of intrashell screening, OR Hubbard manifolds often acquire lower first-principle *U* values,^19^ making the correction even more surgical. Importantly, however, the case of β-NiUO_4_ demonstrates that the improvements of OR-DFT+*U* with respect to shell-averaged schemes are mainly rooted in the exclusion of hybridized states from the Hubbard manifold, and only to a lesser extent (if any) in the reduction of the *U* values.

In closing, the results of the this work suggest that studying the ground state of structurally complex compounds with distorted polyhedra, mixed valence, and hybridized electronic states requires a chemically informed and orbitally resolved framework, which both OR-DFT+*U* and DFT+*U*(WF) provide. A priority for future methodological advancements should be the development of nonempirical, quantitative protocols for identifying Hubbard manifolds (for a given class of projectors) with minimal user input and without reliance on chemical intuition.

## Supplementary Material



## Notes

Occupation eigenvalues for MnUO_4_ and CoUO_4_, projected density of states (PDOS) plots for all Hubbard manifolds and compounds presented, visualization of the OAO projector orbitals and the eigenstates of *β*-NiUO_4_, and a detailed description of the tool used to visualize Hubbard projector functions in real space (PDF)
