# Supplementary material for: Getting the Manifold Right: The Crucial Role of Orbital Resolution in DFT+U for Mixed d–f Electron Compounds
Source: J Chem Theory Comput. 2026 Jan 7;22(2):1016–29. doi: 10.1021/acs.jctc.5c01406 (PMC12854705; doi:10.1021/acs.jctc.5c01406)
Supplement: Supplementary file 1 [file ct5c01406_si_001.pdf]

Supporting Information

Getting the manifold right: The crucial role of orbital resolution in DFT+U for mixed d–f electron compounds

Kinga Warda,[1] Eric Macke,[2] Iurii Timrov,[3] Lucio Colombi Ciacchi,[2] and Piotr M. Kowalski[4]

[1] *Forschungszentrum Jülich GmbH, Institute of Energy Technologies, Theory and Computation of Energy Materials (IET-3), Jülich 52425, Germany, and Jülich Aachen Research Alliance JARA Energy & Center for Simulation and Data Science (CSD) 52428 Jülich, Germany, and Chair of Theory and Computation of Energy Materials Faculty of Georesources and Materials Engineering RWTH Aachen University, 52062 Aachen, Germany*

[2] *Faculty of Production Engineering, Bremen Center for Computational Materials Science and MAPEX Center for Materials and Processes, Hybrid Materials Interfaces Group, University of Bremen, 28359 Bremen, Germany*

[3] *PSI Center for Scientific Computing, Theory, and Data, Paul Scherrer Institute, 5232 Villigen PSI, Switzerland*

[4] *Forschungszentrum Jülich GmbH, Institute of Energy Technologies, Theory and Computation of Energy Materials (IET-3), Jülich 52425, Germany, and Jülich Aachen Research Alliance JARA Energy & Center for Simulation and Data Science (CSD) 52428 Jülich, Germany*

(Dated: November 21, 2025)

Typeset by REVTEX

I. ADDITIONAL COMPUTATIONAL DETAILS

A. DFT+U(WF) calculations

We report additional details concerning the DFT+U(WF) calculations carried out in Ref. 1, whose results are plotted for comparison in Fig. 4 of the main text of this work and whose electronic structures are shown below in Figs. S3–S6. The Wannierzation of bands was performed using the `pmw.x` tool included in the Quantum ESPRESSO distribution, which generates a set of Wannier functions from AO projectors through a simple subspace alignment procedure[2]. If the states possess sufficient atomic-like character, the resulting WFs closely resemble maximally-localized WFs[3] and their use as projectors leads to (almost) integer occupancies. In Ref. 1, first, a regular DFT+U calculation was performed using non-orthogonalized atomic orbitals. Then, `pmw.x` was executed to Wannierize the A-3d and U-4f bands, thus creating new (Wannier-type) projector orbitals. Finally, another SCF calculation was performed in which these new projector orbitals were employed. The resulting occupation patterns as well as the energy differences between the $Ibmm$ and $Cmmm$ structures can be found in the SI of Ref. 1. Note that in the mentioned study the Hubbard U parameters were not recomputed within the Wannier basis; instead, the same LR-cDFT parameters obtained for the non-orthogonalized atomic orbitals were applied in the DFT+U(WF) calculations, too.

B. HSE06 calculations

Given its high efficiency in hybrid calculations, we used the VASP code[4,5] v. 5.4.4 to obtain PDOS plots on the HSE06 level (shown in Figure S6). We employed PAW-type pseudopotentials, considering semicore p states of the A-site cations as explicit valence states and applying a wave function cutoff of 400 eV. The Brilloin zone was sampled using a Γ-centered Monkhorst-Pack grid of dimensions $2 \times 2 \times 2$. All structures were fully relaxed until the forces acting on the ions fell below 0.01 eV. The occupations were broadened using Gaussian smearing with a smearing width of 0.05 eV.

II. ADDITIONAL DATA ON OCCUPATIONS AND ELECTRONIC STRUCTURES OF AUO_4 COMPOUNDS

TABLE S1: Eigenvalues λ corresponding to respective eigenstates ν for different atomic species in $MnUO_4$ and $CoUO_4$, obtained from preliminary PBEsol+U calculations. Hubbard U parameters were set to $U_{\text{Mn-3}d} = 2.0$ eV, $U_{\text{Co-3}d} = 4.0$ eV, $U_{\text{U-5}f} = 2.0$ eV, and $U_{\text{O-2}p} = 1.0$ eV.

System	Atom	Spin	Eigenvalue						
			λ_1	λ_2	λ_3	λ_4	λ_5	λ_6	λ_7
$MnUO_4$	U	↑	0.035	0.109	0.196	0.270	0.302	0.326	0.445
		↓	0.029	0.087	0.113	0.133	0.187	0.228	0.362
	Mn	↑	0.889	0.966	0.987	0.990	0.993	-	-
		↓	0.030	0.047	0.050	0.116	0.165	-	-
	O_1	↑	0.730	0.764	0.822	-	-	-	-
		↓	0.767	0.779	0.817	-	-	-	-
	O_2	↑	0.736	0.775	0.801	-	-	-	-
		↓	0.750	0.792	0.803	-	-	-	-
$CoUO_4$	U	↑	0.063	0.102	0.177	0.200	0.257	0.285	0.425
		↓	0.060	0.089	0.154	0.165	0.200	0.235	0.384
	Co	↑	0.952	0.986	0.989	0.990	0.997	-	-
		↓	0.057	0.126	0.192	0.964	0.980	-	-
	O_1	↑	0.746	0.768	0.818	-	-	-	-
		↓	0.751	0.770	0.806	-	-	-	-
	O_2	↑	0.739	0.792	0.800	-	-	-	-
		↓	0.736	0.774	0.795	-	-	-	-

TABLE S2: Self-consistent Hubbard U parameters obtained via linear-response constrained DFT (LR-cDFT) for alternative setup (3) with an extended Hubbard manifold of U-5$f_{\nu_1-\nu_5}$.

System	U (eV)			
	A-$\widetilde{t_{2g}}$	U-5$f_{\nu_1-\nu_5}$	O1-2p_x	O2-2p_y
$NiUO_4$	7.80	1.51	4.14	4.15
$MnUO_4$	1.50	1.09	4.24	4.17
$CoUO_4$	2.60	1.44	4.10	4.16

TABLE S3: Self-consistent Hubbard U parameters obtained via linear-response constrained DFT (LR-cDFT) for setup (3). The converged values shown here correspond to iteration 2 and demonstrate that the parameters are effectively stable (for comparison with the initial iteration-1 values, see Table 2 in the main text).

System	U (eV)			
	A-$\widetilde{t_{2g}}$	U-5$f_{\nu_1-\nu_4}$	O1-2p_x	O2-2p_y
$MnUO_4$	1.50	0.99	4.25	4.17
$CoUO_4$	2.39	1.24	4.09	4.14

TABLE S4: Difference in total energies between the (experimentally stable) *Ibmm* phases and the more symmetric *Cmmm* ones. The dagger (†) symbolizes that the *Ibmm* structures of $MnUO_4$ and $CoUO_4$ self-relax into *Cmmm*, i.e., there is no stable *Ibmm* structure for these compounds in setup (1). The values in columns labeled "w/o E_{Hub}" were obtained by explicitly removing the on-site Hubbard contribution from the DFT+U total energies, isolating the structural energy differences.

	ΔE (kJ/mol)/f.u.					
	Setup (1)	w/o E_{Hub}	Setup (2)	w/o E_{Hub}	Setup (3)	w/o E_{Hub}
β-$NiUO_4$	-2.75	+0.25	-1.62	-5.10	-1.88	-4.42
$MnUO_4$	†	-	+1.28	-3.18	+0.27	-0.88
$CoUO_4$	†	-	-2.38	-6.19	-1.57	-4.04

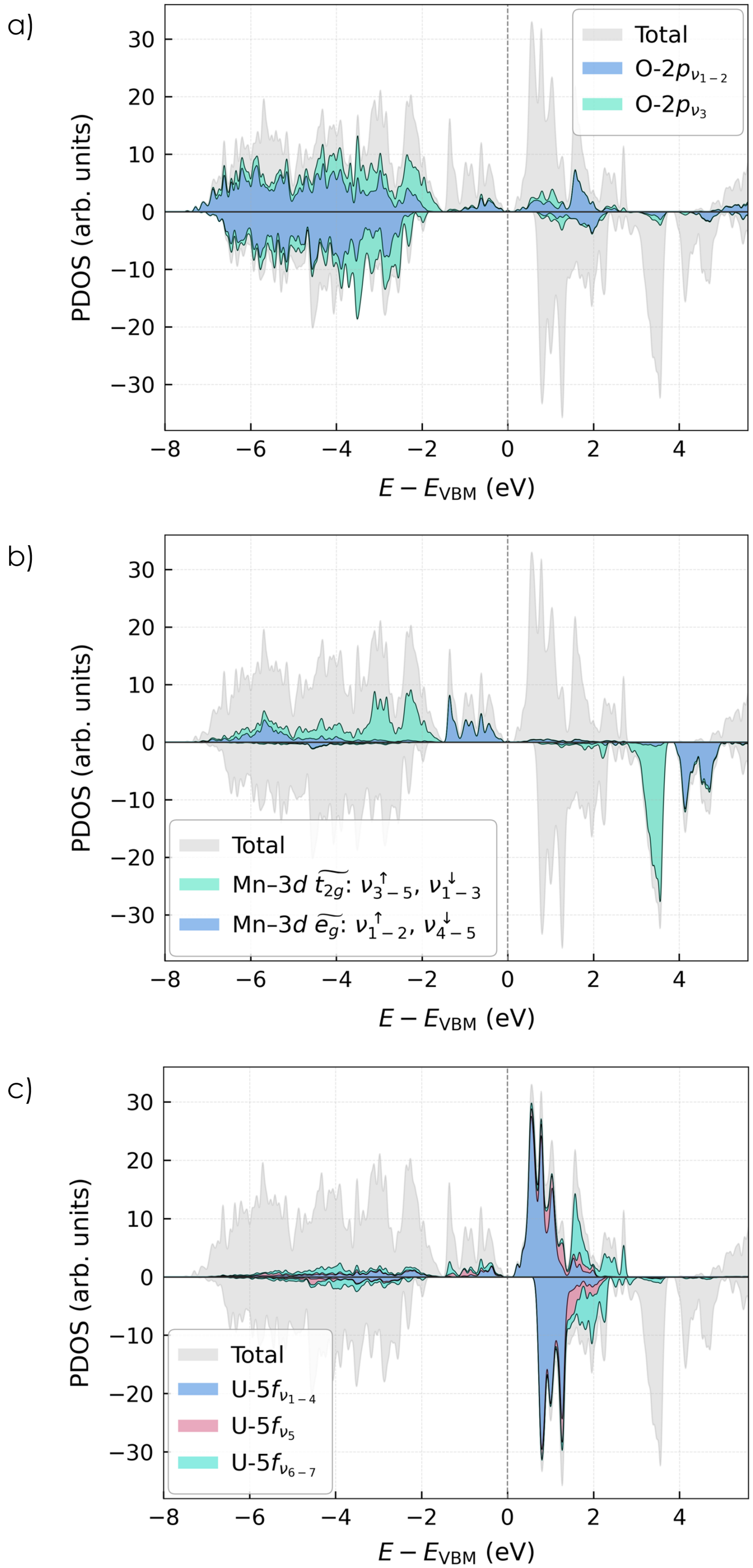

FIG. S1: Stacked PDOS for O-2p (a), Mn-3d (b), and U-5f (c) orbitals in $MnUO_4$, obtained from PBEsol+U calculations with $U_{\text{U-5}f} = 2.0$ eV, $U_{\text{O-2}p} = 1.0$ eV and $U_{\text{Mn-3}d} = 2.0$ eV.

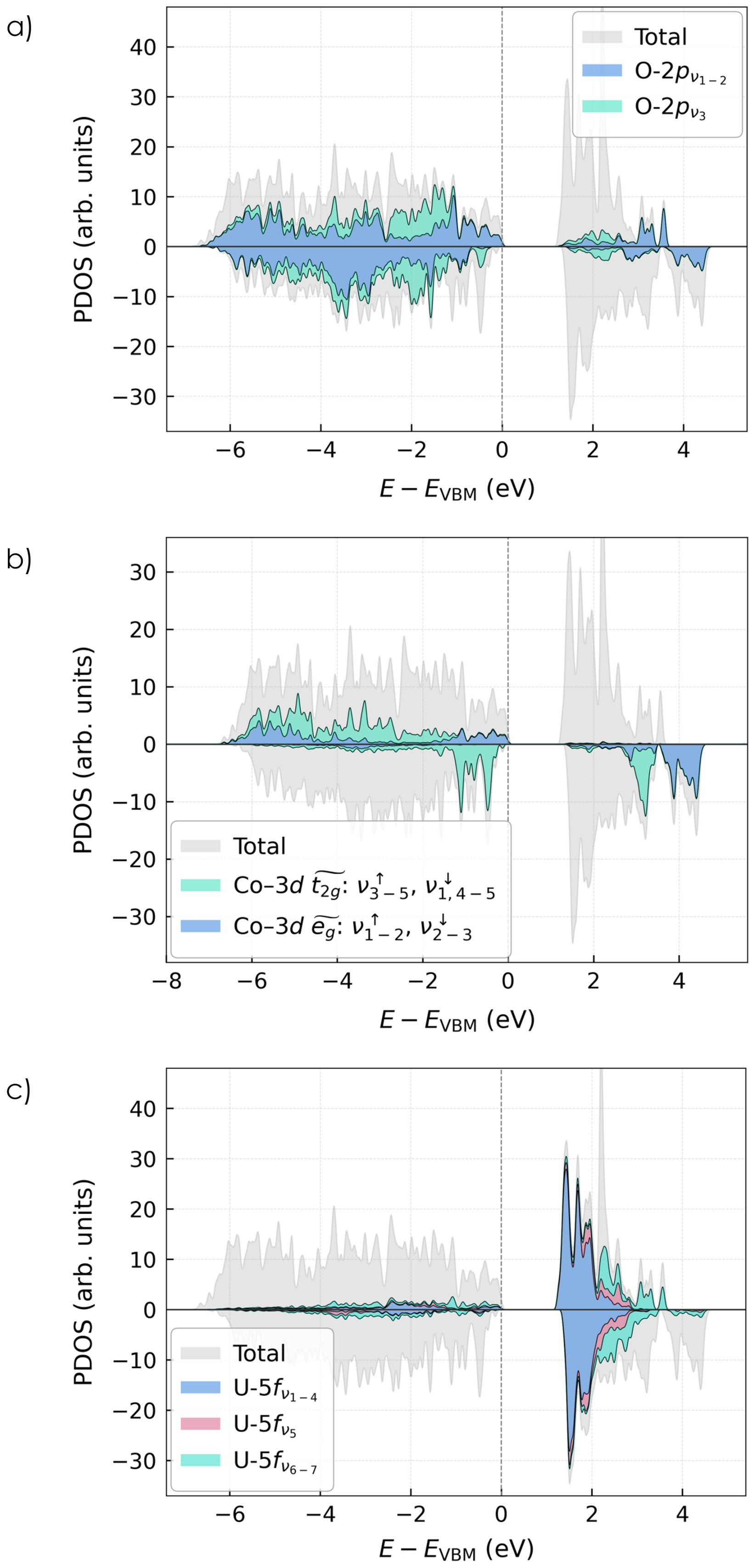

FIG. S2: Stacked PDOS for O-2p (a), Co-3d (b), and U-5f (c) orbitals in $CoUO_4$, obtained from PBEsol+U calculations with $U_{\text{U-5}f} = 2.0$ eV, $U_{\text{O-2}p} = 1.0$ eV and $U_{\text{Co-3}d} = 4.0$ eV.

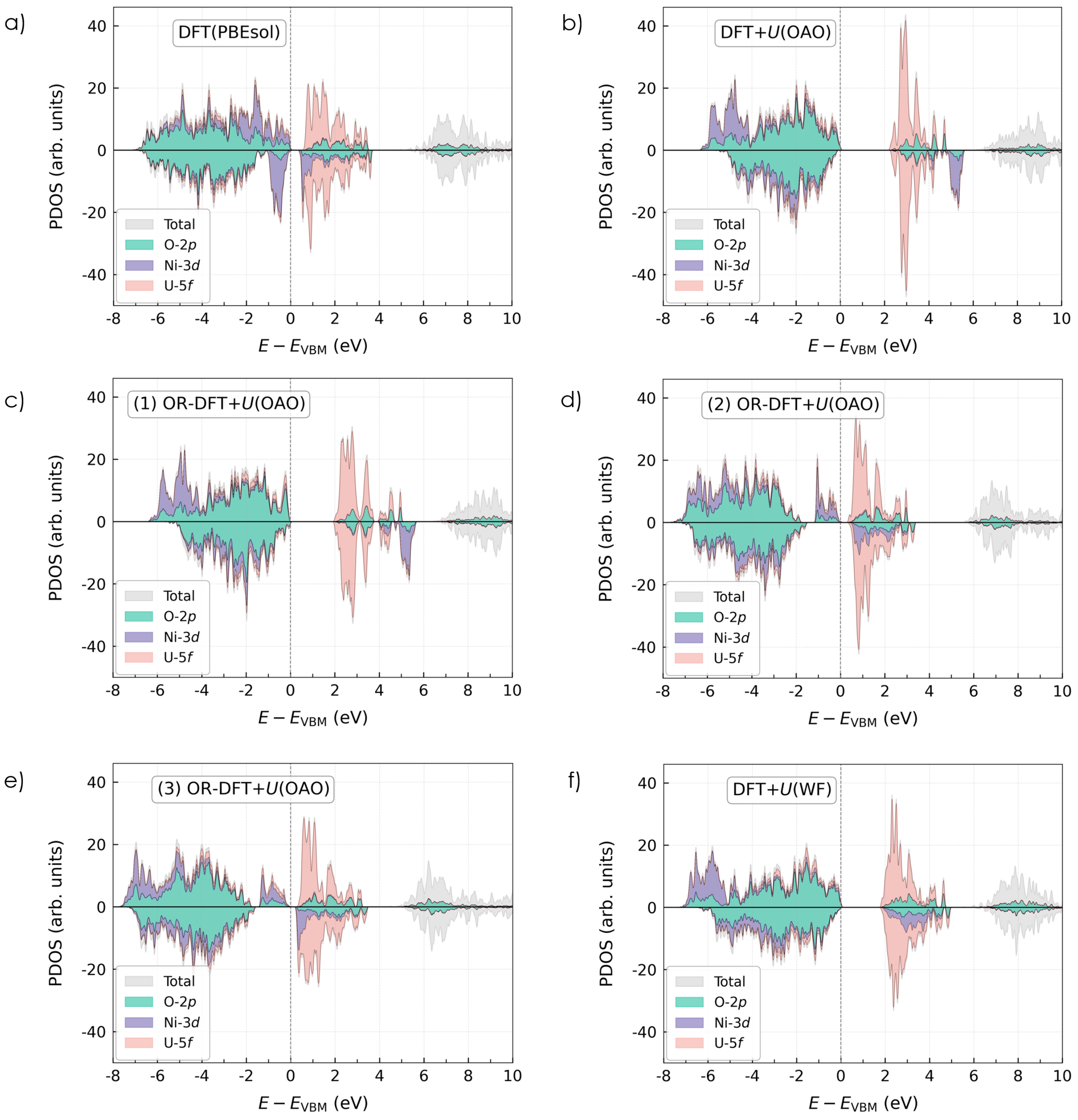

FIG. S3: Stacked PDOS for $NiUO_4$, obtained from (a) PBEsol, (b) PBEsol+U(OAO), (c-e) three orbital-resolved (OR) schemes (see main text for definition) and DFT+U(WF) reproduced here using structural data and computational procedure of Murphy *et al.*[1].

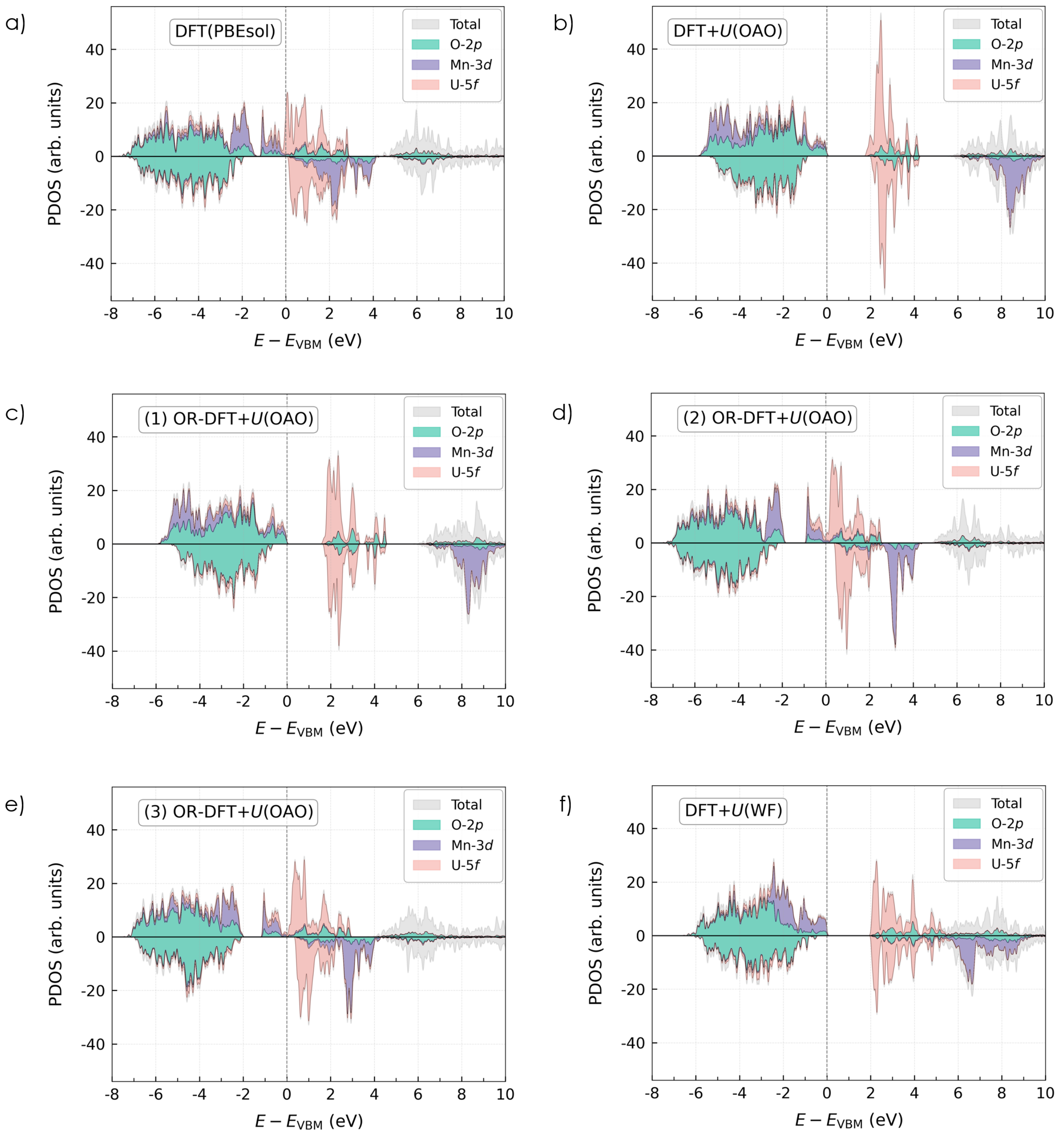

FIG. S4: Stacked PDOS for $MnUO_4$, obtained from (a) PBEsol, (b) PBEsol+U(OAO), (c-e) three orbital-resolved (OR) schemes (see main text for definition) and DFT+U(WF) reproduced here using structural data and computational procedure of Murphy *et al.*[1].

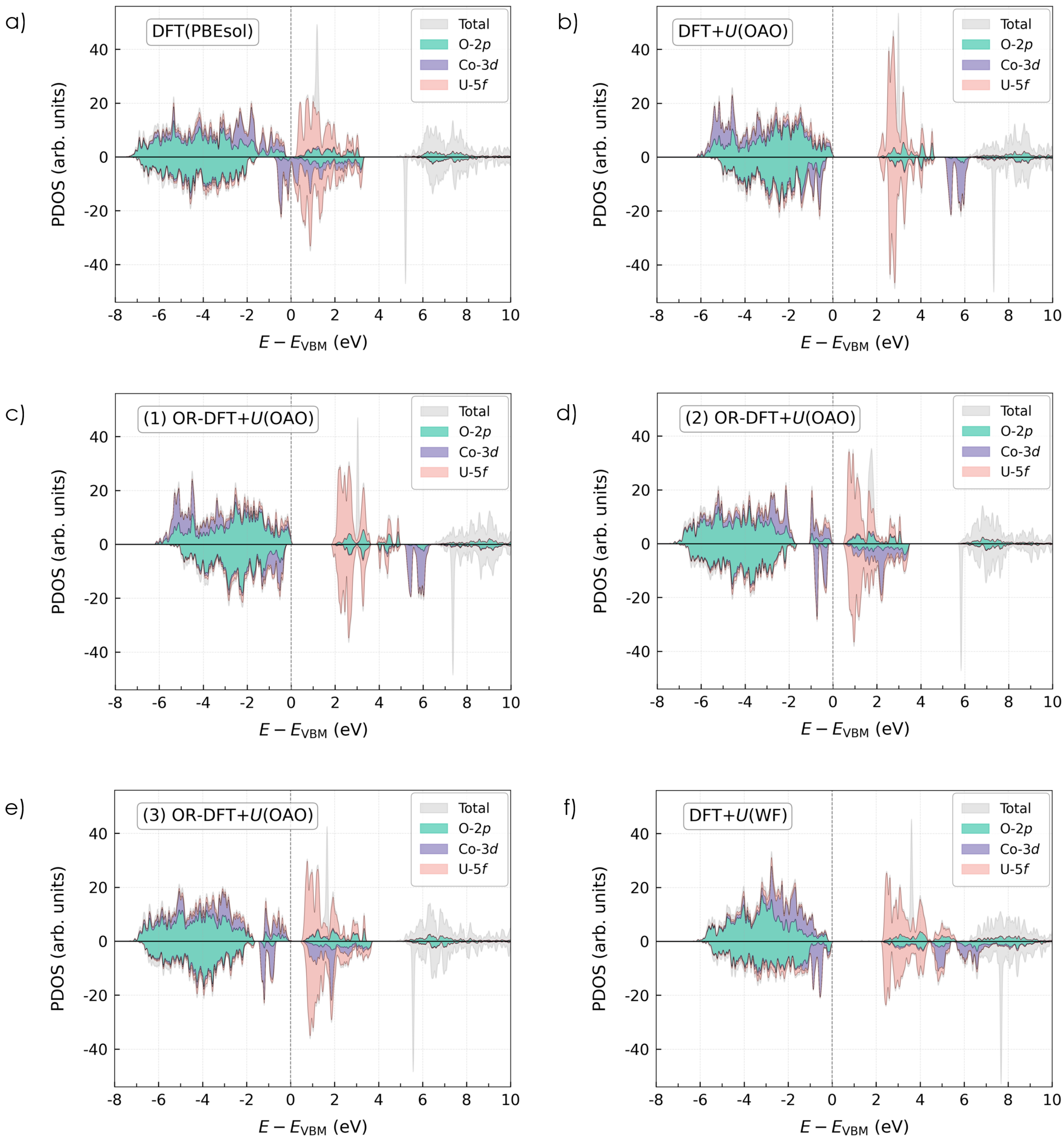

FIG. S5: Stacked PDOS for $CoUO_4$, obtained from (a) PBEsol, (b) PBEsol+U(OAO), (c-e) three orbital-resolved (OR) schemes (see main text for definition) and DFT+U(WF) reproduced here using structural data and computational procedure of Murphy *et al.*[1].

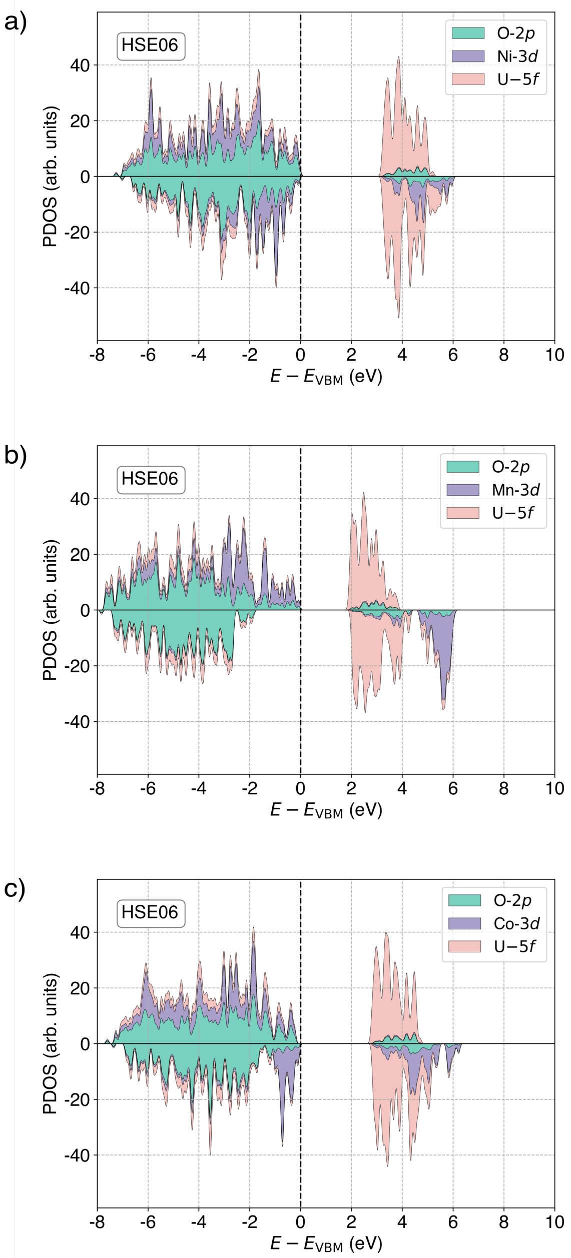

FIG. S6: Stacked PDOS of (a) $NiUO_4$, (b) $MnUO_4$, and (c) $CoUO_4$ obtained from HSE06 calculations performed with VASP.

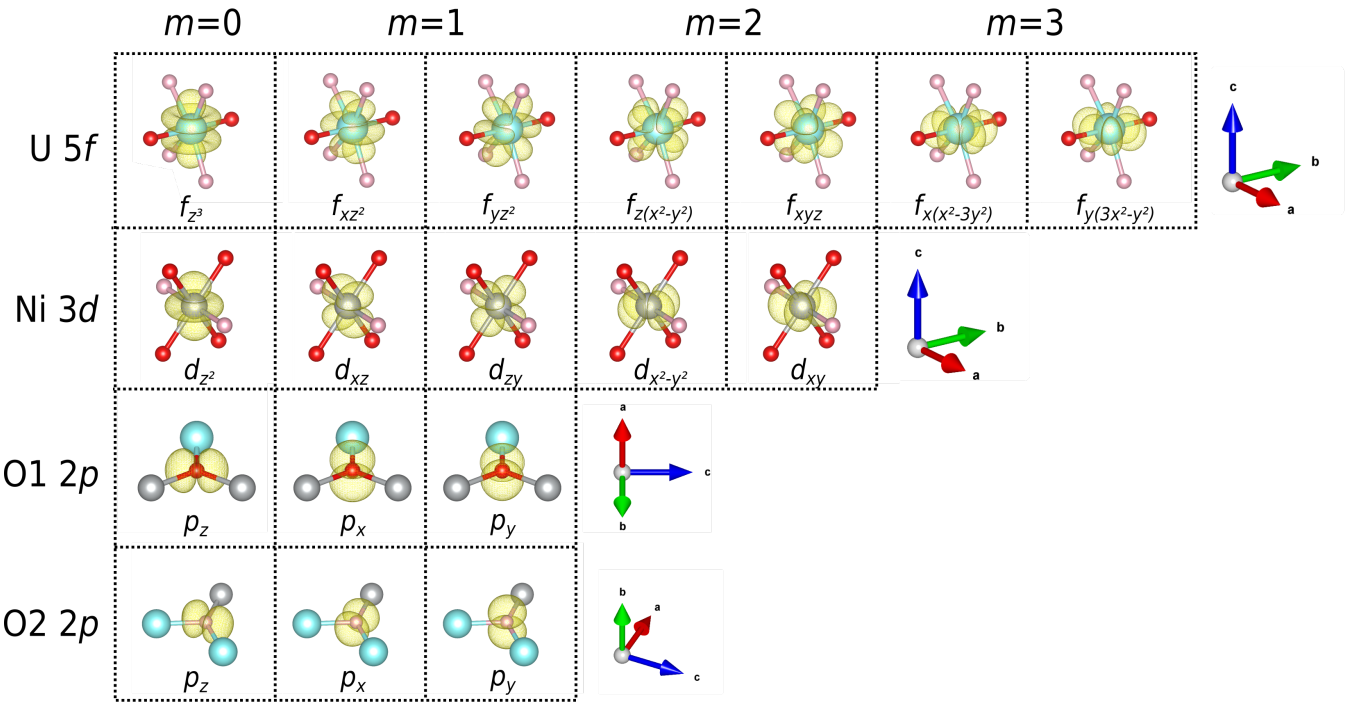

FIG. S7: Orthogonalized atomic orbitals used as Hubbard projectors in DFT+U calculations of $NiUO_4$, plotted using the tool described in Section III. Color code: U – turquoise, Ni – grey, O1 – red, O2 – pink. Note that the orbital labels correspond to the global reference frame of the crystal structure, whereas all other analyses in this work were performed within the local reference frame(s) of the octahedra, as indicated in Sec. 2.1 of the manuscript.

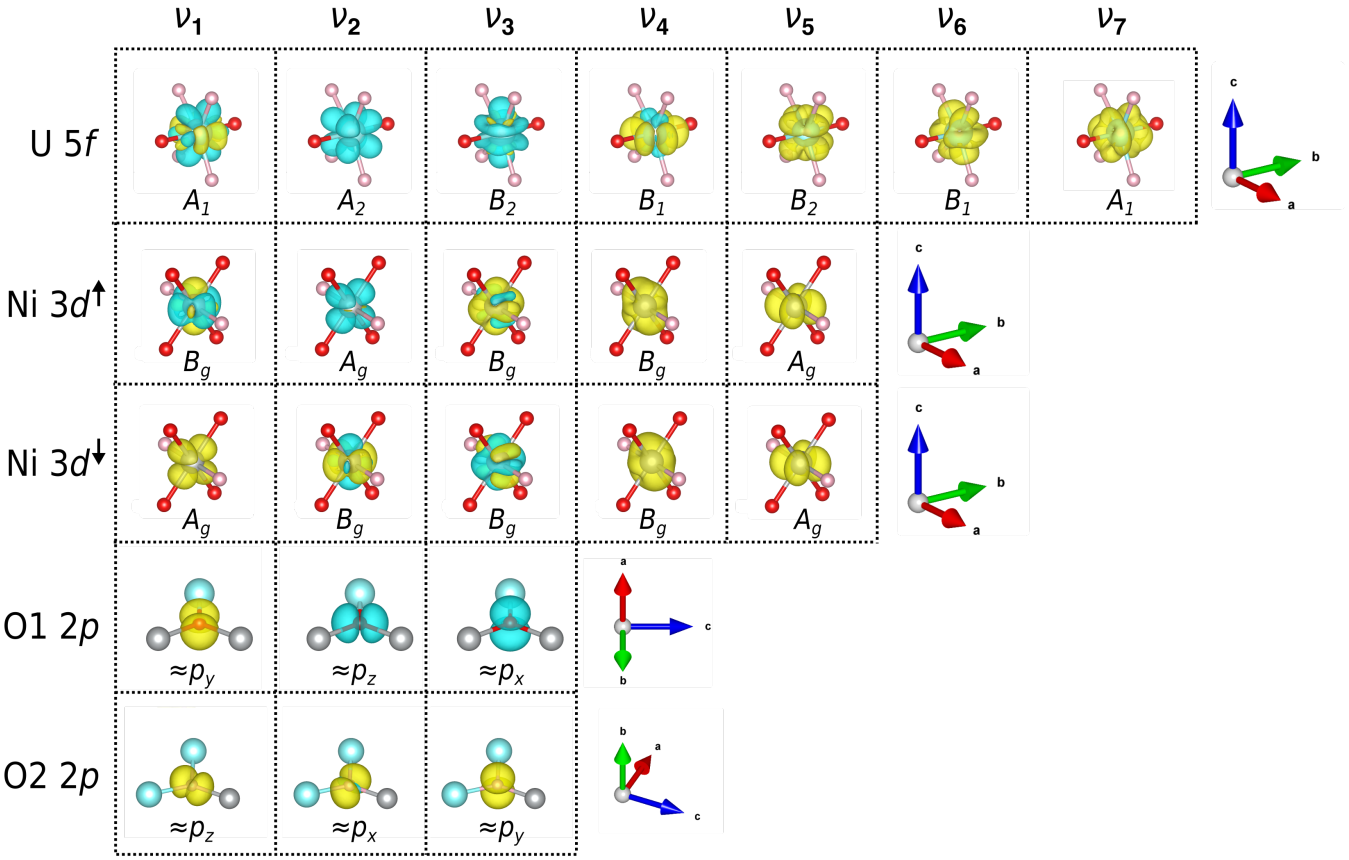

FIG. S8: Eigenstates $\nu_i^{I\sigma}$ of the converged Hubbard occupation matrix $\mathbf{n}$ for $NiUO_4$ (see Section 3.1 of the main text). Each eigenstate is shown as an isosurface of its expansion in the orthogonalized atomic projectors (Fig. S7); yellow (blue) shading denotes positive (negative) coefficients. The corresponding irreducible representation of each state is indicated in its panel. For the U-5f, O1-2p, and O2-2p manifolds, the spin-down eigenstates closely resemble the spin-up ones and have been omitted for clarity.

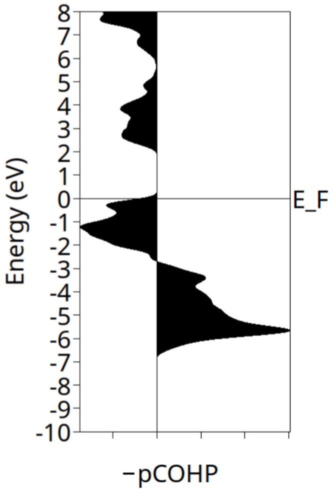

FIG. S9: Projected crystal orbital Hamilton population (pCOHP)[6] averaged over the Ni−O bonds in $NiUO_4$, calculated at the DFT+U(OAO) level using the trial U values presented in the main text. Shown is the negative of the pCOHP, for which values to the left of the center line indicate antibonding interactions (i.e., all states above $\approx -2.5\,\text{eV}$), whereas values to the right represent bonding interactions.

III. POST-PROCESSING TOOL FOR VISUALIZING HUBBARD PROJECTOR FUNCTIONS IN QUANTUM ESPRESSO

In DFT+U calculations, two essential ingredients must be specified beforehand: (i) the value of the Hubbard U parameter, and (ii) the choice of localized Hubbard projector functions, onto which the Kohn–Sham wavefunctions are projected. These projector functions are denoted as $|\varphi^I_m\rangle$ in Eq. (3) of the main text, where I labels the atom and m is the magnetic quantum number associated with a given orbital angular momentum. In coordinate representation, the projector functions are written as $\varphi^I_m(\mathbf{r}-\mathbf{R})$, where $\mathbf{R}$ denotes the unit cell lattice vector. In Quantum ESPRESSO[7–9], several types of Hubbard projectors are available: non-orthogonal atomic orbitals, Löwdin-orthogonalized atomic orbitals[10], and Wannier functions obtained either via a simplified scheme[11] or using the Wannier90 code[12,13]. Given the range of available choices, it is highly beneficial to be able to visualize the Hubbard projector functions for a specific system in order to understand their differences. Each type of projector functions has its own advantages and disadvantages, and the computed physical properties can be quite sensitive to this choice. Therefore, the ability to visualize and compare different projector functions is crucial for interpreting and validating DFT+U results.

To enable the visualization of Hubbard projector functions, we implemented a new feature in the `pp.x` postprocessing tool of Quantum ESPRESSO for plotting the squared modulus of these functions, i.e. $|\varphi^I_m(\mathbf{r}-\mathbf{R})|^2$. The workflow begins with a DFT+U calculation, which writes several data files to disk, including the lattice-periodic parts of the Bloch sums of the Hubbard projector functions in reciprocal space, $\phi^I_{m,\mathbf{k}}(\mathbf{G})$. To enable this output, the user must set the input parameter `disk_io = 'medium'` in the `pw.x` input file. Given that `pp.x` already includes many options for plotting various DFT quantities (e.g., charge density, potentials, magnetization, etc.), the new functionality is accessed via the keyword `plot_num = 25`. The main routine responsible for building the `pp.x` executable is `postproc.f90`, which now calls a newly implemented routine, `hubbard_projectors.f90`, to handle the following steps:

1. Reading reciprocal-space data: The routine reads the lattice-periodic parts of the Bloch sums $\phi^I_{m,\mathbf{k}}(\mathbf{G})$ at each $\mathbf{k}$-point in reciprocal space.

2. Fourier transformation: A Fast Fourier Transform (FFT) is applied to transform $\phi^I_{m,\mathbf{k}}(\mathbf{G})$ into real space, yielding $\phi^I_{m,\mathbf{k}}(\mathbf{r})$.

3. Reconstructing full Bloch functions: As Quantum ESPRESSO handles only the lattice-periodic components of wavefunctions, a phase factor $e^{i\mathbf{k}\cdot\mathbf{r}}$ is applied to reconstruct the full Bloch functions: $\varphi^I_{m,\mathbf{k}}(\mathbf{r}) = e^{i\mathbf{k}\cdot\mathbf{r}}\phi^I_{m,\mathbf{k}}(\mathbf{r})$.

4. Reconstructing real-space localized projectors: A summation over $\mathbf{k}$-points with appropriate phase factors is performed to obtain the localized Hubbard projectors: $\varphi^I_m(\mathbf{r}-\mathbf{R}) = N_{\mathbf{k}}^{-1}\sum_{\mathbf{k}} e^{-i\mathbf{k}\cdot\mathbf{R}}\varphi^I_{m,\mathbf{k}}(\mathbf{r})$, where $N_{\mathbf{k}}$ is the number of $\mathbf{k}$-points. In practice, the unit cell is placed at the origin, so $\mathbf{R}=\mathbf{0}$.

5. Output and visualization: The squared modulus $|\varphi^I_m(\mathbf{r}-\mathbf{R})|^2$ is computed and written to disk. This output can then be visualized using external tools such as XCrySDen[14] or VESTA[15].

When visualizing Hubbard projector functions for atoms near the cell boundaries in small unit cells, the functions may appear truncated. To address this, our implementation allows the user to define a virtual supercell (based on the unit cell) and to apply a rigid shift of the function within the supercell (e.g., toward its center) ensuring that the functions are not clipped by cell boundaries. A working example is provided as example #08 in the `PP/examples` directory of the Quantum ESPRESSO distribution, demonstrating how to use this feature. Figure S10 illustrates the differences between Hubbard projector functions constructed with and without Löwdin orthogonalization. When inter-atomic orthogonalization is applied, the originally atomic-like orbitals lose their pure character, hybridizing with ligand states and avoiding spatial overlap with nearby ligand ions.

---

[1] Gabriel L. Murphy, Zhaoming Zhang, Rebekka Tesch, Piotr M. Kowalski, Maxim Avdeev, Eugenia Y. Kuo, Daniel J. Gregg, Philip Kegler, Evgeny V. Alekseev, and Brendan J. Kennedy, "Tilting and Distortion in Rutile-Related Mixed Metal Ternary Uranium Oxides: A Structural, Spectroscopic, and Theoretical Investigation," Inorg. Chem. **60**, 2246–2260 (2021).

[2] Stefano Fabris, Stefano De Gironcoli, Stefano Baroni, Gianpaolo Vicario, and Gabriele Balducci, "Taming multiple valency with density functionals: A case study of defective ceria," Physical Review B **71**, 041102 (2005).

[3] Nicola Marzari and David Vanderbilt, "Maximally localized generalized Wannier functions for composite energy bands," Physical Review B **56**, 12847–12865 (1997).

[4] G. Kresse and J. Furthmüller, "Efficiency of ab-initio total energy calculations for metals and semiconductors using a plane-wave basis set," Computational Materials Science **6**, 15–50 (1996).

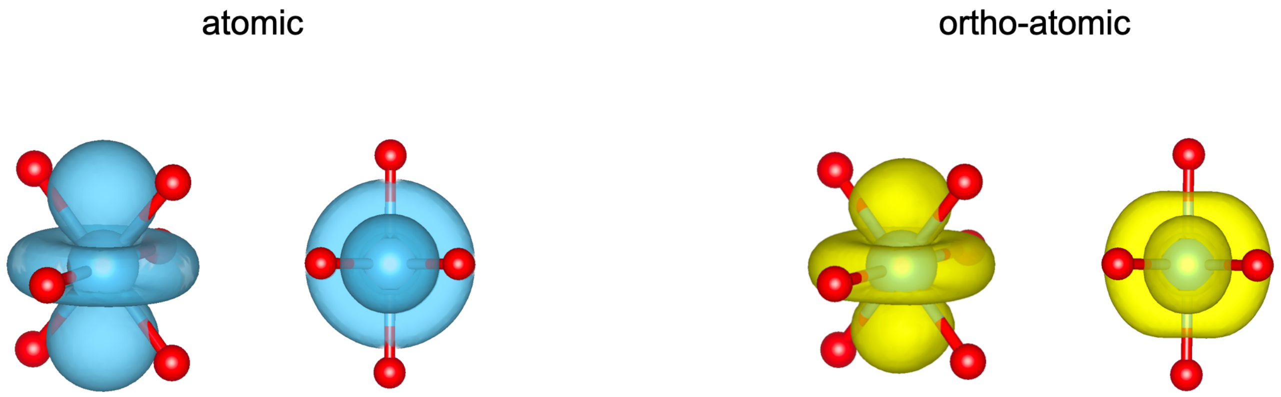

FIG. S10: (Left) Visualization of the atomic d_{z^2} orbital used as one of the basis functions for the Hubbard projectors (corresponding to the `'atomic'` keyword). (Right) Visualization of the d_{z^2}-like orbital obtained via Löwdin orthogonalization (corresponding to the `'ortho-atomic'` keyword). Red spheres represent oxygen atoms, and the blue sphere corresponds to a 3d transition-metal atom. For illustrative purposes, the TiO_2 system was used to generate these images.

[5] G. Kresse and J. Furthmüller, "Efficient iterative schemes for *ab initio* total-energy calculations using a plane-wave basis set," Physical Review B **54**, 11169–11186 (1996).

[6] Stefan Maintz, Volker L. Deringer, Andrei L. Tchougréeff, and Richard Dronskowski, "LOBSTER: A tool to extract chemical bonding from plane-wave based DFT," Journal of Computational Chemistry **37**, 1030–1035 (2016).

[7] Paolo Giannozzi, Stefano Baroni, Nicola Bonini, Matteo Calandra, Roberto Car, Carlo Cavazzoni, Davide Ceresoli, Guido L Chiarotti, Matteo Cococcioni, Ismaila Dabo, Andrea Dal Corso, Stefano De Gironcoli, Stefano Fabris, Guido Fratesi, Ralph Gebauer, Uwe Gerstmann, Christos Gougoussis, Anton Kokalj, Michele Lazzeri, Layla Martin-Samos, Nicola Marzari, Francesco Mauri, Riccardo Mazzarello, Stefano Paolini, Alfredo Pasquarello, Lorenzo Paulatto, Carlo Sbraccia, Sandro Scandolo, Gabriele Sclauzero, Ari P Seitsonen, Alexander Smogunov, Paolo Umari, and Renata M Wentzcovitch, "QUANTUM ESPRESSO: A modular and open-source software project for quantum simulations of materials," J. Phys.: Condens. Matter **21**, 395502 (2009).

[8] P Giannozzi, O Andreussi, T Brumme, O Bunau, M Buongiorno Nardelli, M Calandra, R Car, C Cavazzoni, D Ceresoli, M Cococcioni, N Colonna, I Carnimeo, A Dal Corso, S De Gironcoli, P Delugas, R A DiStasio, A Ferretti, A Floris, G Fratesi, G Fugallo, R Gebauer, U Gerstmann, F Giustino, T Gorni, J Jia, M Kawamura, H-Y Ko, A Kokalj, E Küçükbenli, M Lazzeri, M Marsili, N Marzari, F Mauri, N L Nguyen, H-V Nguyen, A Otero-de-la-Roza, L Paulatto, S Poncé, D Rocca, R Sabatini, B Santra, M Schlipf, A P Seitsonen, A Smogunov, I Timrov, T Thonhauser, P Umari, N Vast, X Wu, and S Baroni, "Advanced capabilities for materials modelling with Quantum ESPRESSO," J. Phys.: Condens. Matter **29**, 465901 (2017).

[9] P. Giannozzi, O. Baseggio, P. Bonfà, D. Brunato, R. Car, I. Carnimeo, C. Cavazzoni, S. de Gironcoli, P. Delugas, F. Ferrari Ruffino, A. Ferretti, N. Marzari, I. Timrov, A. Urru, and S. Baroni, "Quantum ESPRESSO toward the exascale," J. Chem. Phys. **152**, 154105 (2020).

[10] Per-Olov Löwdin, "On the Non-Orthogonality Problem Connected with the Use of Atomic Wave Functions in the Theory of Molecules and Crystals," J. Chem. Phys. **18**, 365–375 (1950).

[11] S. Fabris, S. de Gironcoli, S. Baroni, G. Vicario, and G. Balducci, "Taming multiple valency with density functionals: A case study of defective ceria," Phys. Rev. B **71**, 041102(R) (2005).

[12] Giovanni Pizzi, Valerio Vitale, Ryotaro Arita, Stefan Blügel, Frank Freimuth, Guillaume Géranton, Marco Gibertini, Dominik Gresch, Charles Johnson, Takashi Koretsune, Julen Ibañez-Azpiroz, Hyungjun Lee, Jae-Mo Lihm, Daniel Marchand, Antimo Marrazzo, Yuriy Mokrousov, Jamal I Mustafa, Yoshiro Nohara, Yusuke Nomura, Lorenzo Paulatto, Samuel Poncé, Thomas Ponweiser, Junfeng Qiao, Florian Thöle, Stepan S Tsirkin, Małgorzata Wierzbowska, Nicola Marzari, David Vanderbilt, Ivo Souza, Arash A Mostofi, and Jonathan R Yates, "Wannier90 as a community code: New features and applications," J. Phys.: Condens. Matter **32**, 165902 (2020).

[13] Alberto Carta, Iurii Timrov, Peter Mlkvik, Alexander Hampel, and Claude Ederer, "Explicit demonstration of the equivalence between DFT+U and the Hartree-Fock limit of DFT+DMFT," Phys. Rev. Res. **7** (2025), 10.1103/physrevresearch.7.013289.

[14] T. Kokalj, "XCrySDen–a new program for displaying crystalline structures and electron densities," J. Mol. Graphics Modelling **17**, 176 (1999).

[15] K. Momma and F. Izumi, "VESTA: a three-dimensional visualization system for electronic and structural analysis," J. Appl.

Crystallogr. **41**, 653 (2008).
